# Comprehensive transcriptome profiling of BET inhibitor-treated HepG2 cells

**DOI:** 10.1371/journal.pone.0266966

**Published:** 2022-04-29

**Authors:** Mina Baek, Jin Choul Chai, Hae In Choi, Eunyoung Yoo, Bert Binas, Young Seek Lee, Kyoung Hwa Jung, Young Gyu Chai

**Affiliations:** 1 Department of Molecular and Life Science, Hanyang University, Ansan, Republic of Korea; 2 Institute of Natural Science and Technology, Hanyang University, Ansan, Republic of Korea; 3 College of Veterinary Medicine, Seoul National University, Seoul, Republic of Korea; 4 Department of Bionanotechnology, Hanyang University, Seoul, Republic of Korea; 5 Department of Biopharmaceutical System, Gwangmyeong Convergence Technology Campus of Korea Polytechnic II, Incheon, Republic of Korea; Texas Tech Health Sciences Center El Paso, UNITED STATES

## Abstract

Hepatocellular carcinoma (HCC) is the most common primary liver cancer and poor prognosis. Emerging evidence suggests that epigenetic alterations play a crucial role in HCC, suggesting epigenetic inhibition as a promising therapeutic approach. Indeed, the bromodomain and extra-terminal (BET) inhibitors inhibit the proliferation and invasion of various cancers but still lack a strong mechanistic rationale. Here, we identified the differentially expressed mRNAs (DEmRNAs) and lncRNAs (DElncRNAs) in human HCC cell line HepG2 treated with the BET inhibitors, JQ1, OTX015, or ABBV-075. We analyzed the correlation between DEmRNAs and DElncRNAs in common for the three inhibitors based on their expression profiles and performed functional annotation pathway enrichment analysis. Most of these shared DEmRNAs and DElncRNAs, including some novel transcripts, were downregulated, indicating decreased proliferation/adhesion and increased apoptosis/inflammation. Our study suggests that BET proteins play a crucial role in regulating cancer progression-related genes and provide a valuable resource for novel putative biomarkers and therapeutic targets in HCC.

## Introduction

Hepatocellular carcinoma (HCC) is the most common primary liver cancer [1]. Chronic liver disease and cirrhosis are the most important risk factors for developing HCC. Emerging evidence suggests that the accumulation of genetic and epigenetic aberrations is associated with the initiation and progression of HCC. The multi-targeted tyrosine kinase inhibitor sorafenib is currently being used as the first-line treatment for HCC [2–4].

The bromodomain and extra-terminal (BET) family of epigenetic reader proteins is comprised of bromodomain-containing protein 2 (BRD2), BRD3, BRD4 and BRDT that bind to acetylated residues on histones and transcription factors in the enhancer or promoter region of genes [5]. BET family proteins regulate the expression of oncogenes and have become potential therapeutic targets for cancer [6, 7]. Indeed, BET inhibitors, such as JQ1, OTX015, I-BET151, and ABBV-075 inhibit the proliferation and invasion of a range of cancers [8]. JQ1 has been extensively studied as the first identified BET inhibitor, and JQ1 treatment significantly reduces cell proliferation and induces apoptosis in several cancer types. In the case of HCC, JQ1 suppresses HCC tumorigenesis by reducing the expression of active-enhancer-related genes such as the oncogene c-MYC and the cell cycle regulatory molecule E2F2 [9–11]. OTX015 was the first BET inhibitor to be tested in a clinical trial. It exhibited antitumor activity against leukemia, lymphoma, nuclear protein in testis (NUT) carcinoma, and other solid cancers [12–14]. ABBV-075, a recently developed oral pan-BET inhibitor, has been reported to induce apoptosis and tumor regression in several hematological and solid cancer types [15].

Long non-coding RNAs (lncRNAs) are RNA transcripts longer than 200 nucleotides that are not translated into proteins [16]. They play crucial roles in gene regulation, including chromatin remodeling, transcription, and post-transcriptional regulation [17]. Importantly, lncRNAs are emerging as key regulators of cancer signaling [18]; they are aberrantly expressed in various cancers and play critical roles in tumor initiation, progression, and metastasis, suggesting clinical potential as biomarkers and therapeutic targets [19–21]. To date, a total of 74 lncRNAs have been reported to be involved in HCC, including *HULC* [22], *ATB* [23], *HOTTIP* [24], *HOTAIR* [25], and *ZEB1-AS1* [26], the expression of which is deregulated in HCC [27]. HCC-associated lncRNAs are significantly associated with tumor clinicopathological and metastatic features and can have either oncogenic or tumor-suppressive properties. They also affect several cancer phenotypes by altering miRNA and mRNA expression and stability [27, 28].

The present study performed a comprehensive analysis of mRNA and lncRNAs expression profiles of BET inhibitor-treated human HCC cell line HepG2 using RNA sequencing (RNA-Seq). We identified differentially expressed mRNAs (DEmRNAs) and lncRNAs (DElncRNAs) and analyzed the correlation between DEmRNAs and DElncRNAs. By focusing on the transcripts commonly up- and downregulated by JQ1, OTX015, and ABBV-075, we identified potentially significant transcripts for HCC progression, including some novel DEmRNAs and DElncRNAs.

## Materials and methods

### Cell culture and treatment

HCC cell lines HepG2 and Huh7 were purchased from Korean Cell Line Bank (Seoul, Korea). The cells were cultured in MEM containing 10% heat-inactivated FBS, penicillin, and streptomycin (Thermo Fisher Scientific, Waltham, MA, USA). They were maintained at 37°C in a humidified atmosphere with 95% air/5% CO_2_. The BET inhibitors, JQ1, OTX015, and ABBV-075, were purchased from Tocris Bioscience (Minneapolis, MN, USA). They were dissolved in dimethyl sulfoxide (DMSO) at a stock concentration of 10 mM and stored at -20°C. The cells were treated with various concentrations (50 nM, 500 nM, and 5μM) of JQ1, OTX015, or ABBV-075 for different durations (12 h and 24 h).

### Cell viability assay

Cell viability was examined using a PreMix water-soluble tetrazolium-1 (WST-1) cell proliferation assay kit (Takara, Shiga, Japan). The HepG2 cells were seeded at a density of 5 x 10^3^ per well in 96-well plates and treated with JQ1, OTX015, or ABBV-075 for 0 to 24 h. Thereafter, PreMix WST-1 was added to each well and the incubation was continued at 37°C for another 4 h. Absorbance was measured using a microplate reader at 450 nm. The data represent three independent experiments (n = 3).

### RNA library preparation and RNA-seq

Total RNA was extracted from 4 groups (12 independent samples) samples. Experimental triplicates of HepG2 cells were treated with DMSO (3 samples), 5 μM JQ1 (3 samples), or 5 μM OTX015 (3 samples) for 24 h or with 50 nM ABBV-075 (3 samples) for 12 h. The RNA library was created as previously described [29]. Briefly, total RNA was extracted using RNAiso Plus (Takara, Shiga, Japan) and the RNeasy Mini Kit (QIAGEN Inc., Hilden, Germany) according to the manufacturer’s instructions. Ribosomal RNA (rRNA) was depleted using RiboMinus Eukaryote kit (Invitrogen, Carlsbad, CA, USA). RNA libraries were created using the NEBNext Ultra Directional RNA Library Preparation Kit for Illumina (New England BioLabs, Ipswich, MA, USA). Transcriptome sequencing was performed using the Illumina HiSeq2500 platform (Macrogen, Seoul, Korea).

### Differentially expressed mRNAs and lncRNAs analysis

The DEmRNAs and DElncRNAs were analyzed following a previously reported pipeline [30]. Briefly, the FASTQC file was checked for quality control, and adapters and low-quality bases were removed using Trimmomatic [31]. The FASTQ files were aligned to the *Homo sapiens* reference sequence GRCh38 using STAR (version 2.7.1) software [32]. DESeq2 [33] was used with the default parameters to obtain DEmRNAs and DElncRNAs. DESeq2 used the median method to normalize sequencing depth and RNA composition. Fold change in expression ≥ 1.5 log_2_ and adjusted *p*-value (*p*adj) ≤ 0.05 were used as the criteria to define DEmRNAs and DElncRNAs. The RNA-seq data were deposited in the Gene Expression Omnibus database under dataset accession numbers GSE158552 and GSE189376.

### Correlation analysis

The Pearson correlation coefficient (PCC) and *R* value were calculated to assess the similarity of the gene expression profiles. Then the correlation coefficients between DEmRNAs and DElncRNAs were weighted by a power function. We selected the PCC greater than 0.8 as the meaningful value.

### Functional annotation and canonical pathway analysis

Database for Annotation, Visualization, and Integrated Discovery (DAVID version 6.8, http://david.abcc.ncifcrf.gov/home.jsp) was used to determine functions most significantly enriched in the genes in the datasets [34]. DAVID uses a modified Fisher’s exact *p-*value to examine gene ontology (GO) enrichment. A *p-*value ≤ 0.05 was used as the criterion for GO term analysis. Values less than 0.05 are considered to indicate enrichment in the annotation category. KEGG Orthology-Based Annotation System (KOBAS version 3.0, http://kobas.cbi.pku.edu.cn/) was used to analyze the annotation and identification of enriched KEGG pathways [35]. All DEmRNAs were subjected to Gene-list Enrichment analysis under default conditions. Enriched *p*-value ≤ 0.05 was used as the criterion for KOBAS. Genomic Regions Enrichment of Annotations Tool (GREAT) was used for functional annotation analysis of DElncRNAs [36]. GREAT analyzes the annotations of nearby genes to assign biological associations to sets of non-coding genomic regions. GREAT analysis was performed by submitting the chromosomal locations of differentially expressed DElncRNAs to the website http://bejerano.stanford.edu/great/public/html/. A *p*-value ≤ 0.05 was used as the criterion for GREAT analysis. Gene Set Enrichment Analysis (GSEA) software (http://www.broad.mit.edu/gsea/msigdb/) was used to analyze the biological processes of statistically significant gene sets [37]. All DEmRNAs with normalized counts were submitted to the GSEA and analyzed with gene symbol annotation in default conditions. GSEA calculated a normalized enrichment score (NES) for the expression level of each gene set. A *p*-value ≤ 0.05 was used as the criterion for GSEA. Each of these genes was mapped to objects by Ingenuity Pathway Analysis [38] (IPA; QIAGEN Inc., Hilden, Germany, https://www.qiagenbioinformatics.com/products/ingenuitypathway-analysis). IPA software conducts functional analysis to show genes involved in biological functions and disease.

### Total RNA extraction and quantitative RT-PCR

Total RNA extractions and cDNA sample preparation were performed using Takara’s kits according to the manufacture’s instruction (Takara, Shiga, Japan). Quantitative reverse transcription PCR (qRT-PCR) was performed using an ABI 7500 real-time PCR system (Applied Biosystems Inc., Foster City, CA, USA). The critical threshold (ΔCT) values were normalized to glyceraldehyde-3-phosphate dehydrogenase (GAPDH) expression for mRNAs and U6 for lncRNAs, which served as an internal control. The results were also analyzed using the comparative critical threshold (ΔΔCT) method. Specific primers were designed using Primer Bank (http://pga.mgh.harvard.edu/primerbank/index.html). The primers for qRT-PCR are listed in the S1 Table.

### Statistical analyses

The statistical analyses were performed using IBM SPSS Statistics ver. 26.0 (IBM Corporation, Armonk, NY, USA). All data are expressed as the mean ± standard deviation (SD). All qRT-PCR data were tested using one-way ANOVA followed by Tukey’s honestly significant difference (HSD) post hoc test. Differences for which p < 0.05 were considered significant.

## Results

### Effects of BET inhibitor on HCC cell line HepG2

**Fig 1:**
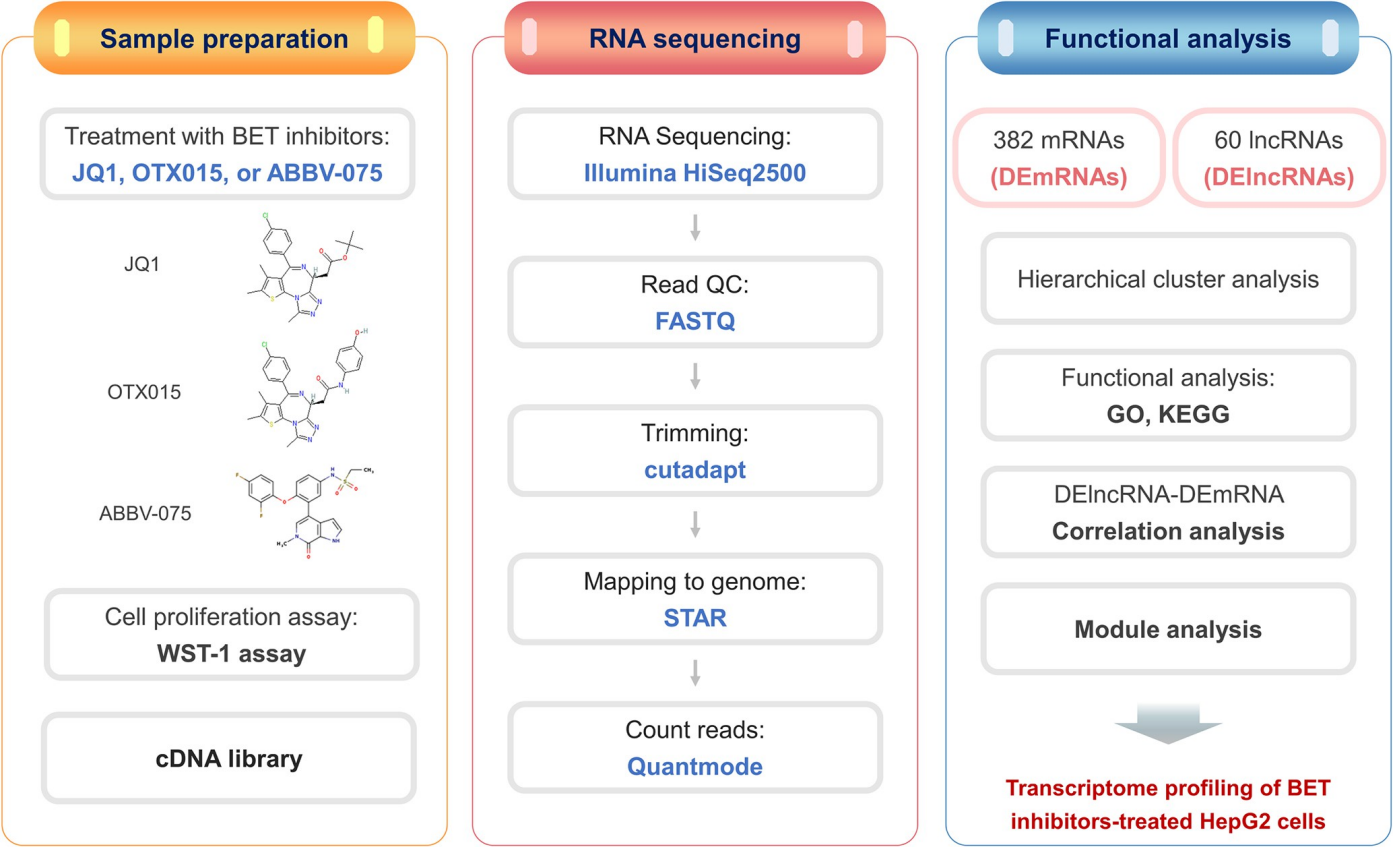
Flow chart of bioinformatics analysis of DEmRNAs and DElncRNAs. DEmRNAs; differentially expressed mRNAs, DElncRNAs; differentially expressed lncRNAs, GO; gene ontology, KEGG; kyoto encyclopedia of genes and genomes.

The flowchart for the comprehensive analysis of DEmRNAs and DElncRNAs is shown in Fig 1. We first determined the optimal experimental conditions for transcriptional profiling. We have previously shown with EdU incorporation assays that BET inhibitors reduce HepG2 cell proliferation [39]. We now examined their effects on cell viability at various concentrations (50 nM, 500 nM, and 5 μM) and time points (12 h and 24 h). JQ1, OTX015, and ABBV-075 significantly reduced cell proliferation in a dose-dependent manner after 12 h and 24 h (S1 Fig). However, ABBV-075 was effective earlier and at a lower concentration than the other two inhibitors for a given degree of inhibition. For the gene expression profiling, the HepG2 cells were therefore treated with 5 μM of JQ1 or OTX015 for 24 h, but with 50 nM of ABBV-075 for 12 h.

### Identification of differentially expressed mRNAs (DEmRNAs) and lncRNAs (DElncRNAs) in BET inhibitor-treated HepG2 cells

**Fig 2:**
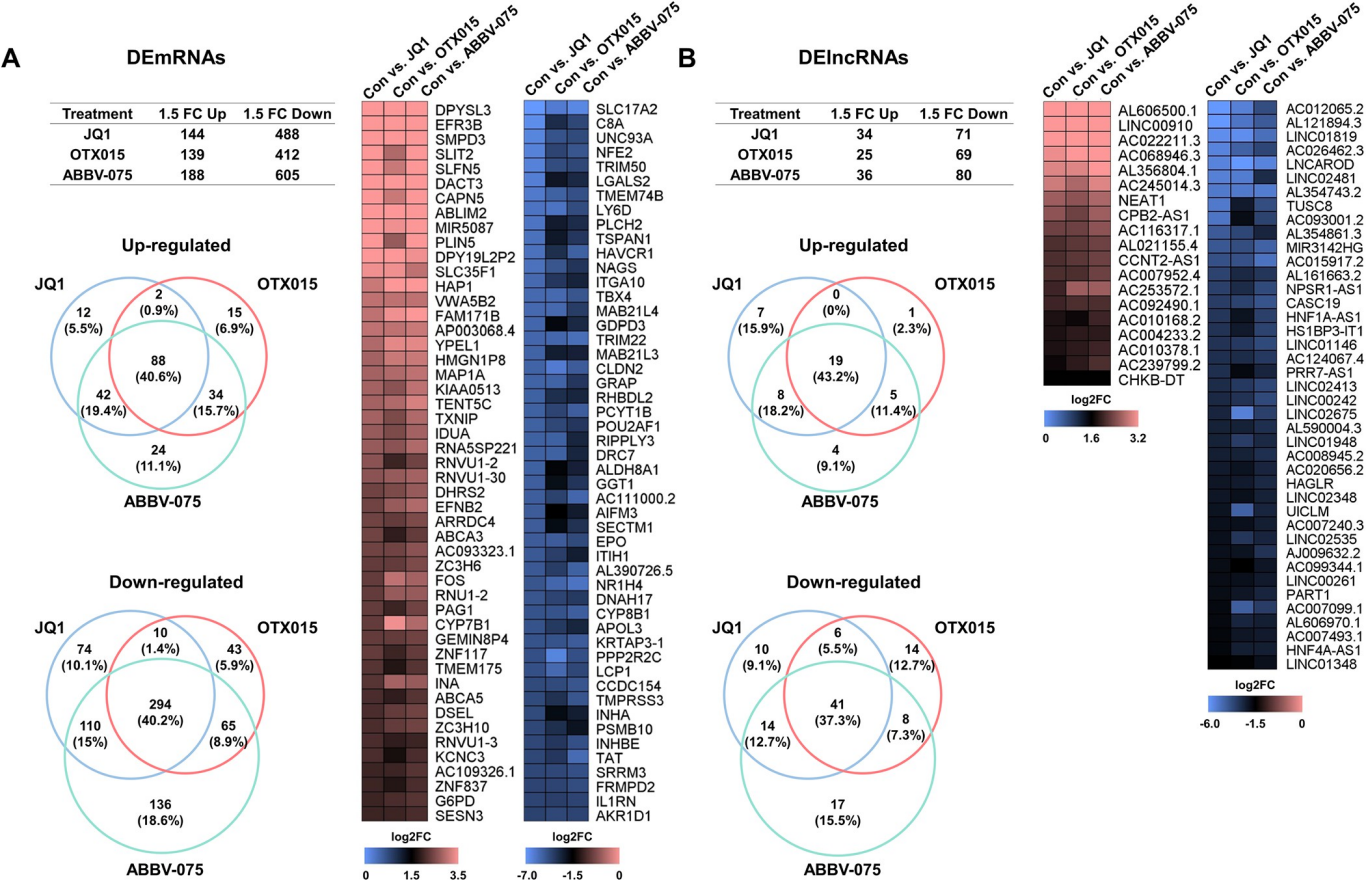
Identification of DEmRNAs and DElncRNAs.

Following cDNA library construction and RNA sequencing (RNA-seq), the DEmRNAs (Fig 2A) and DElncRNAs (Fig 2B) were selected with a cutoff of log_2_ fold change ≥ 1.5, log_2_ fold change ≤ -1.5, and *p*adj ≤ 0.05. In the JQ1-treated HepG2 cells, a total of 632 DEmRNAs (including 144 upregulated and 488 downregulated) and 105 DElncRNAs (including 34 upregulated and 71 downregulated) were identified. In the OTX015-treated HepG2 cells, a total of 551 DEmRNAs (including 139 upregulated and 412 downregulated) and 94 DElncRNAs (including 25 upregulated and 69 downregulated) were identified. In the ABBV-075-treated HepG2 cells, 793 DEmRNAs (including 188 upregulated and 605 downregulated) and 116 DElncRNAs (including 36 upregulated and 80 downregulated) were identified. For all BET inhibitor-treated cells, the up- and downregulated DEmRNAs and DElncRNAs are listed in S2–S7 Tables, respectively. 382 DEmRNAs and 60 DElncRNAs were common for all three inhibitors (S8 and S9 Tables, respectively). The top 50 up- and downregulated DEmRNAs and DElncRNAs are shown as heat maps in Fig 2.

The expression profiles of DEmRNAs and DElncRNAs in HepG2 cells treated with JQ1 or OTX015 for 24 h, or with ABBV-075 for 12 h. The Table displays the number of up- and downregulated DEmRNAs (A) and DElncRNAs (B) of BET inhibitor-treated HepG2 cells. The Venn diagrams show the numbers of overlapping up- and downregulated DEmRNAs (A) and DElncRNAs (B). The heat maps represent the top 50 up- and downregulated DEmRNAs (A) and DElncRNAs (B) (log_2_ fold change ≥ 1.5, log_2_ fold change ≤ -1.5, and *p*adj ≤ 0.05). The color scales represent the log_2_ fold change values. Red cells indicate upregulated genes, blue cells indicate downregulated genes. Each experiment was performed in experimental triplicates (n = 3) for each condition, and the results were individually combined. In the BET inhibitor-treated HepG2 cells, 382 DEmRNAs and 60 DElncRNAs were identified in common.

### Functional annotation and pathway enrichment analysis of commonly expressed DEmRNAs and DElncRNAs treated with BET inhibitors

**Fig 3:**
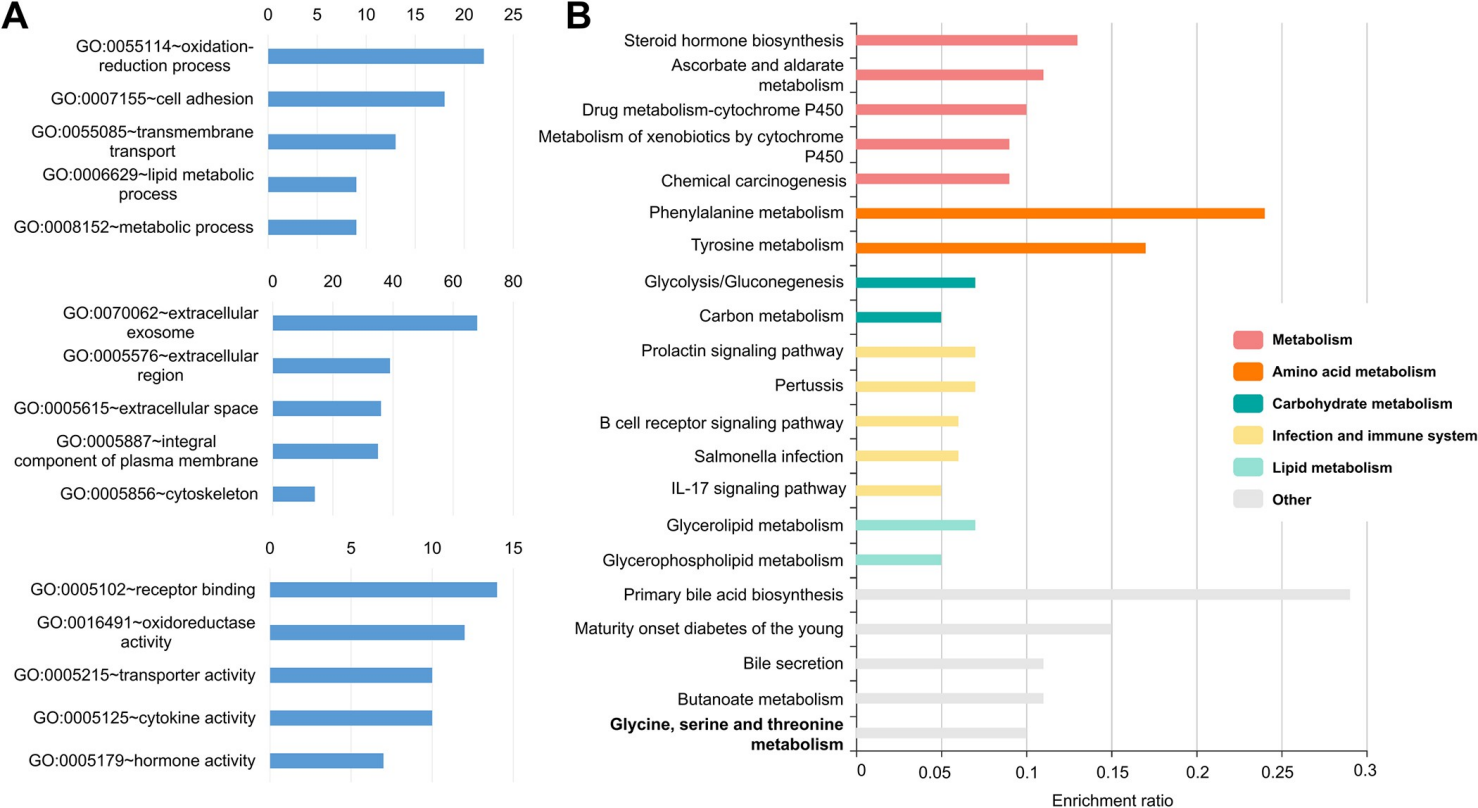
Functional annotation and pathway enrichment analysis of commonly expressed DEmRNAs treated with BET inhibitors. (A) GO term analysis of commonly expressed DEmRNAs. The numbers of genes enriched for each term are displayed for the top 5 GO terms in biological process (BP; upper panel), cellular component (CC; middle panel), and molecular function (MF; bottom panel). (B) KEGG pathway enrichment analysis of commonly expressed DEmRNAs. Each row represents an enriched function, and the length of the bar represents the enrichment ratio, which is calculated as input gene number/background gene number (KOBAS, http://kobas.cbi.pku.edu.cn). In the KOBAS algorithm, clusters are divided according to the values calculated for the enriched terms. The colors of the bars represent different clusters and show the top 5 with the highest enrichment ratios.

We performed GO and KEGG pathway analysis to explore the functional classification and pathway enrichment of DEmRNAs and DElncRNAs commonly up- and downregulated in response to BET inhibitor treatment. GO term analysis categorized the results into the biological process (BP), cellular component (CC), and molecular function (MF) (Fig 3A). Functional annotation analysis of DEmRNAs using DAVID revealed that the most enriched GO terms in the BP category were mainly associated with redox processes, cell adhesion, transmembrane transport, lipid metabolism, and metabolic processes. In CC categories, the DEmRNAs were most enriched in extracellular exosome, extracellular region, an integral component of the plasma membrane, and cytoskeleton. In MF categories, the most significant terms were related to receptor binding, oxidoreductase activity, cytokine activity, transporter activity, and hormone activity. The KEGG pathway analysis for up- and downregulated genes was performed using KOBAS 3.0. The KEGG pathway results (Fig 3B) found primary bile acid biosynthesis, phenylalanine metabolism, taurine, and hypotaurine metabolism, tyrosine metabolism, and maturity-onset diabetes of the young, suggesting that most enriched KEGG pathways were mainly involved in metabolic pathways. We also performed GSEA of DEmRNAs commonly up- and downregulated in response to BET inhibitor treatment (S2A Fig). The degree of enrichment gene sets is shown as a normalized enrichment score (NES). Positive and negative NES values indicate enrichment at the top and bottom of the ranked gene set, respectively. The GSEA approach gave similar results as the GO analysis using DAVID, and the most enriched GO terms were mainly related to metabolic processes. Functional annotation analysis of DElncRNAs using GREAT revealed that DElncRNAs were located near the transcriptional start site, suggesting that the identified DElncRNAs could regulate protein-coding genes (S3A–S3C Fig). We also found that DElncRNAs regulated by the three BET inhibitors are enriched in a number of positive and negative biological processes, metabolic processes, and signaling pathways (S3D Fig).

### Functional annotation and pathway enrichment analysis of DEmRNAs of each BET inhibitor treatment

GO term and KEGG pathway analyses were performed to functionally annotate the genes that were commonly up- and downregulated in each BET inhibitor treatment. The GO analysis revealed that the corresponding BP categories were enriched in negative regulation of cell growth, oxidation-reduction process, cellular response of heparin, and autophagic cell death (S4A–S4C Fig, upper panels). The downregulated DEmRNAs were related to biological processes such as cell adhesion, transmembrane transport, drug response, angiogenesis, and metabolism. The top 4 enriched pathways of the KEGG results are shown in the middle panels of S4 Fig. The KEGG pathway analysis revealed that the upregulated DEmRNAs were significantly enriched in ABC transporters, axon guidance, and parathyroid hormone synthesis, secretion, and action. In contrast, the downregulated DEmRNAs were enriched in metabolic pathways, PI3K-Akt signaling pathway, drug metabolism—cytochrome P450, and bile secretion. Thus, after treatment with different BET inhibitors, the commonly up- and downregulated genes were highly enriched in metabolic processes and pathways. We also performed GSEA of DEmRNAs (bottom panel of S4 Fig) and obtained similar results as in the GO analysis using DAVID, i.e., the DEmRNAs in each BET-inhibitor-treated group were positively correlated with metabolic processes. DEmRNAs in the JQ1-treated group were negatively correlated with genes involved in the regulation of catalytic activity, and the groups treated with OTX015 and ABBV-075 were negatively correlated with genes involved in the cellular response to stress and binding of protein- containing complexes, respectively.

### Analysis of correlations between DElncRNAs and DEmRNAs

**Fig 4:**
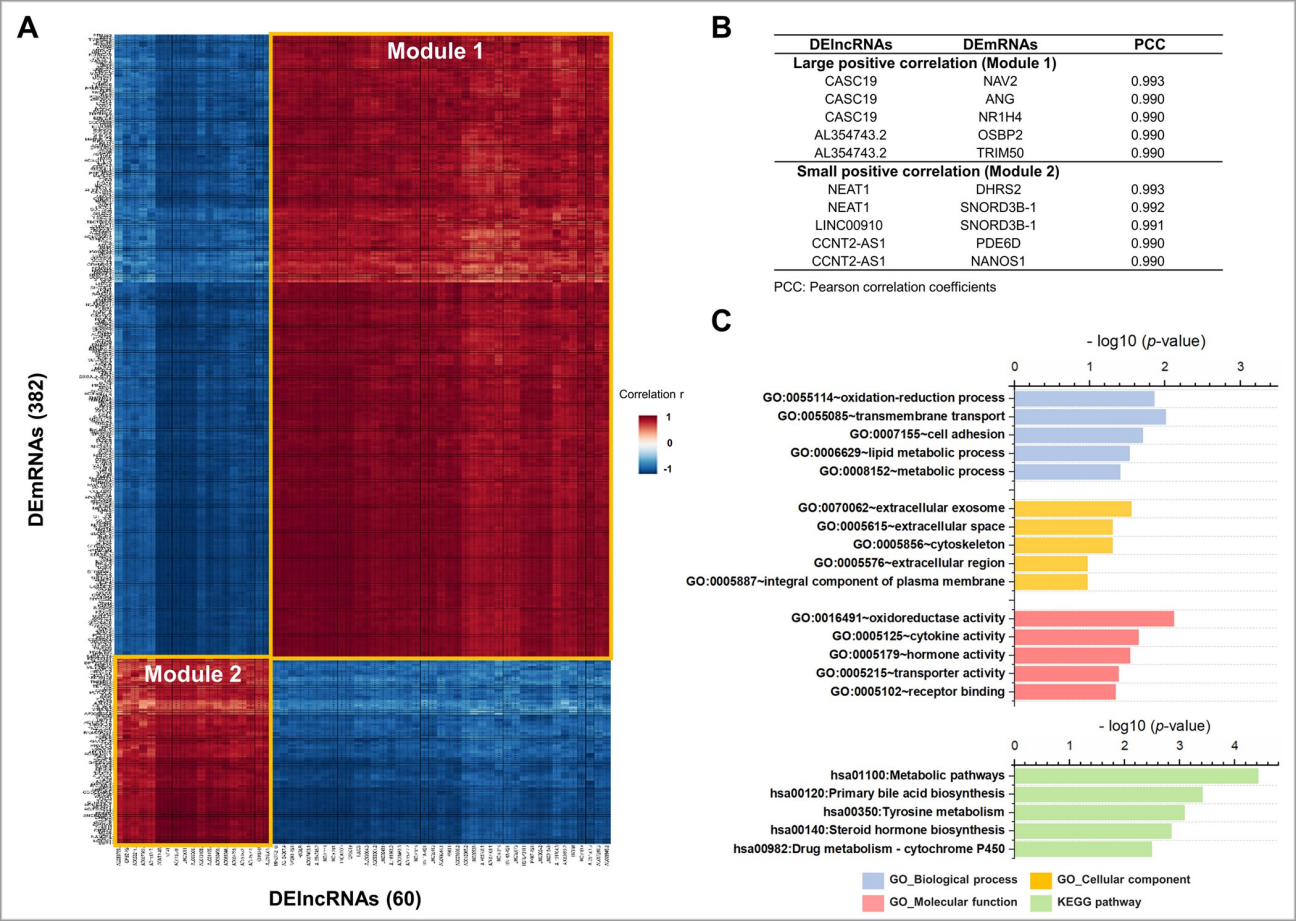
Correlation analysis and functional annotation of DEmRNAs in Module 1. (A) Correlation heat map of the DEmRNAs and DElncRNAs. The color scale shown in the heat map represents the correlation *R* values. Each row represents a DEmRNA, and each column represents a DElncRNA. Pearson correlation coefficients were calculated to determine the relationship of each lncRNA with individual mRNA. Red cells indicate a positive correlation, while blue cells indicate a negative correlation with a higher correlation indicated by higher color intensity as shown by the color scale. (B) Top 5 DElncRNA-DEmRNA pairs with correlation coefficients > 0.985 in HepG2 cells treated with JQ1, OTX015, and ABBV-075 in common. (C) The top 5 GO terms and KEGG pathways for DEmRNAs in Module 1 are shown. GO terms are divided into 3 categories (BP, CC, and MF).

Pearson correlation analysis identified 22,950 DElncRNA-DEmRNA pairs (|*r*| ≥ 0.7, *p-*value ≤ 0.05), which included 382 DEmRNAs and 60 DElncRNAs commons to all BET-treated cells. The Pearson correlation coefficient (PCC) values were visualized by a heat map (Fig 4A). Based on the DElncRNAs and correlated DEmRNAs expression patterns, the nodes and correlations were divided into two main Modules. Among the positively correlated DElncRNA-DEmRNA pairs, the large and small segments were defined as Modules 1 and 2, respectively. Interestingly, the expression of both DEmRNAs and DElncRNAs were downregulated in Module 1, while in Module 2 they were both upregulated. The top 5 DElncRNA-DEmRNA pairs with positive correlation coefficients > 0.985 are shown in Fig 4B. The PCC values of the DElncRNA-DEmRNA pairs in Modules1 and 2 are listed in the S10 and S11 Tables, respectively.

### Analysis of Module 1—Functional annotation and pathway enrichment analysis

Based on the results of correlation analysis, we performed functional annotation of the DEmRNAs of Module 1. The top 5 GO terms of the BP, CC, and MF are shown in Fig 4C. In the BP categories, most enriched GO terms were related to cell adhesion, transmembrane transport, metabolic process, angiogenesis, and response to drugs, similar for each BET inhibitor treatment. In the CC categories, the DEmRNAs were most enriched in integral components of membrane and plasma membrane. In the MF categories, the most significant terms were related to receptor binding, cytokine activity, oxidoreductase activity, transporter activity, and hormone activity. The KEGG pathway results showed phenylalanine metabolism, primary bile acid biosynthesis, taurine and hypotaurine metabolism, and tyrosine metabolism, suggesting that the most enriched KEGG pathways were mainly involved in metabolic pathways. We also performed GSEA of DEmRNAs of Module 1 (S2B Fig). This approach yielded similar results as the GO analysis using DAVID, i.e. the most enriched GO terms were mainly involved in metabolic processes.

### Analysis of Module 1—Biofunctional analysis

**Fig 5:**
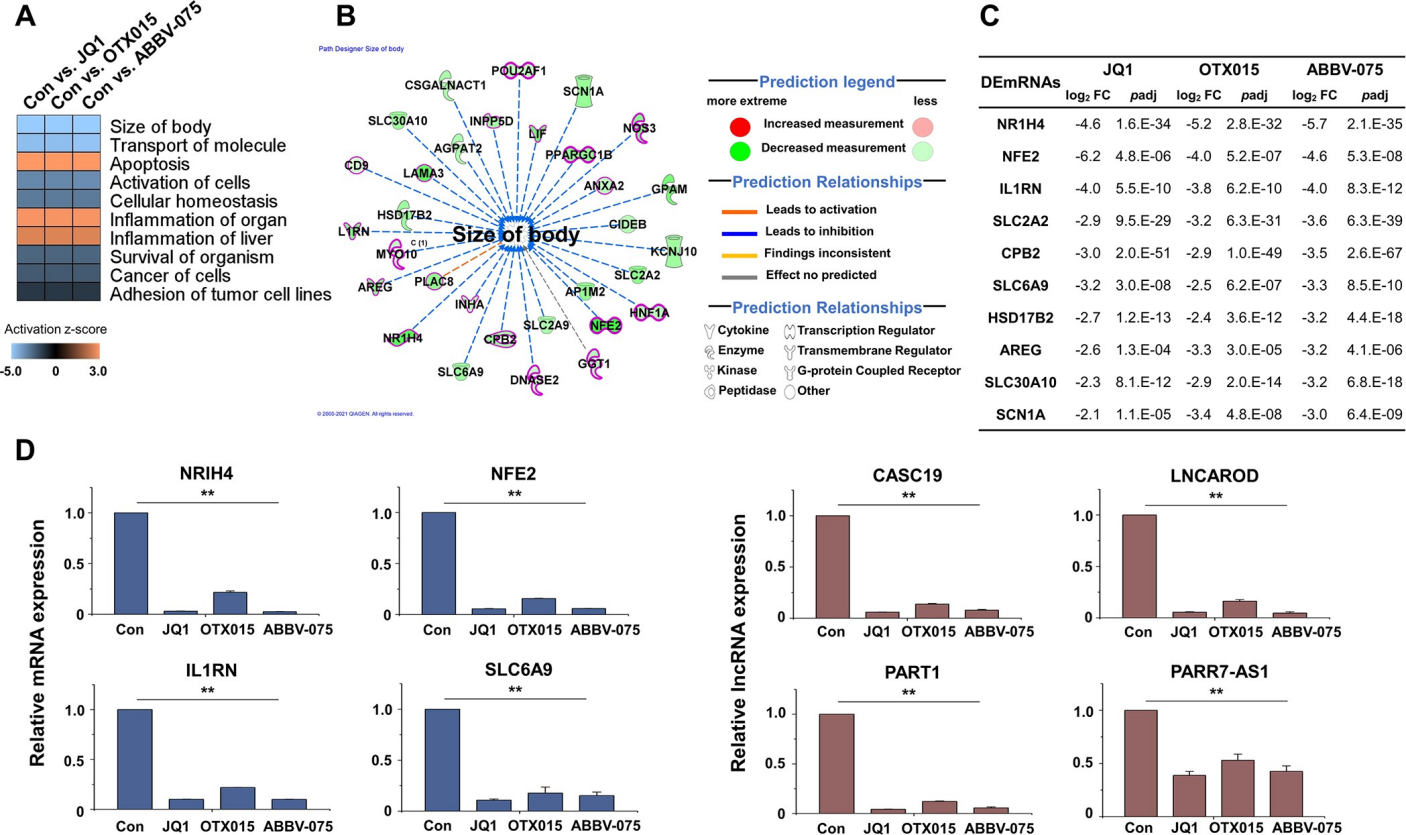
Biofunctional analysis of JQ1-, OTX015-, and ABBV-075-treated HCC cells. (A) Biological pathway analysis of DEmRNAs using IPA for genes of Module 1 (QIAGEN Inc., https://www.qiagenbioinformatics.com/products/ingenuitypathway-analysis). Pink cells indicate that the pathway is activated. In contrast, blue cells indicate that the pathway is inhibited, with higher Z-scores indicated by higher color intensity as shown by the color scale. (B) IPA downstream effects analysis showing the mapping of most significant genes related to the size of the body. Of the genes related to the size of the body, only cellular growth and proliferation-related genes are shown in bold and red. The DEmRNAs are colored by predicted activation state, such as activated (red) or suppressed (green). The edges connecting the nodes are colored orange (leading to activation of the downstream node), blue (leading to inhibition), and yellow (if the findings underlying the relationship are inconsistent with the state of the downstream node). (C) Log_2_ fold changes and *p*adj in the expression of genes related to the size of the body. (D) Expression levels of the size of body-related mRNAs and lncRNAs were analyzed by qRT-PCR and normalized to GAPDH or U6 transcript levels, respectively. Overall, the size of body-related mRNAs and lncRNAs were inhibited by BET inhibitors. The data represent three independent experiments. The values are the mean ± SD of triplicate experiments (**p < 0.01).

To further understand the molecular functions of the genes, we conducted biofunctional analysis using Ingenuity Pathway Analysis (IPA). Activation Z-scores were used to predict whether biological functions are activated or inhibited after treatment with BET inhibitors. Most of the biofunctional results showed inhibition, including the size of the body, transport of molecules, activation of cells, cellular homeostasis, and survival of an organism. In contrast, several biofunctions were activated, including apoptosis, glucose metabolism disorder, and inflammation of organs (Fig 5A). IPA downstream effects analysis identifies biological processes and functions related to the size of the body and predicts whether these processes increase or decrease (Fig 5B). Of the genes related to the size of the body, only the top 10 genes related to cellular growth and proliferation are listed in Fig 5C. To validate the RNA-seq results, we confirmed the expression of the DEmRNAs and DElncRNAs by qRT-PCR (Fig 5D). Several DEmRNAs and DElncRNAs related to the size of the body and its functions were selected and validated.

**Fig 6:**
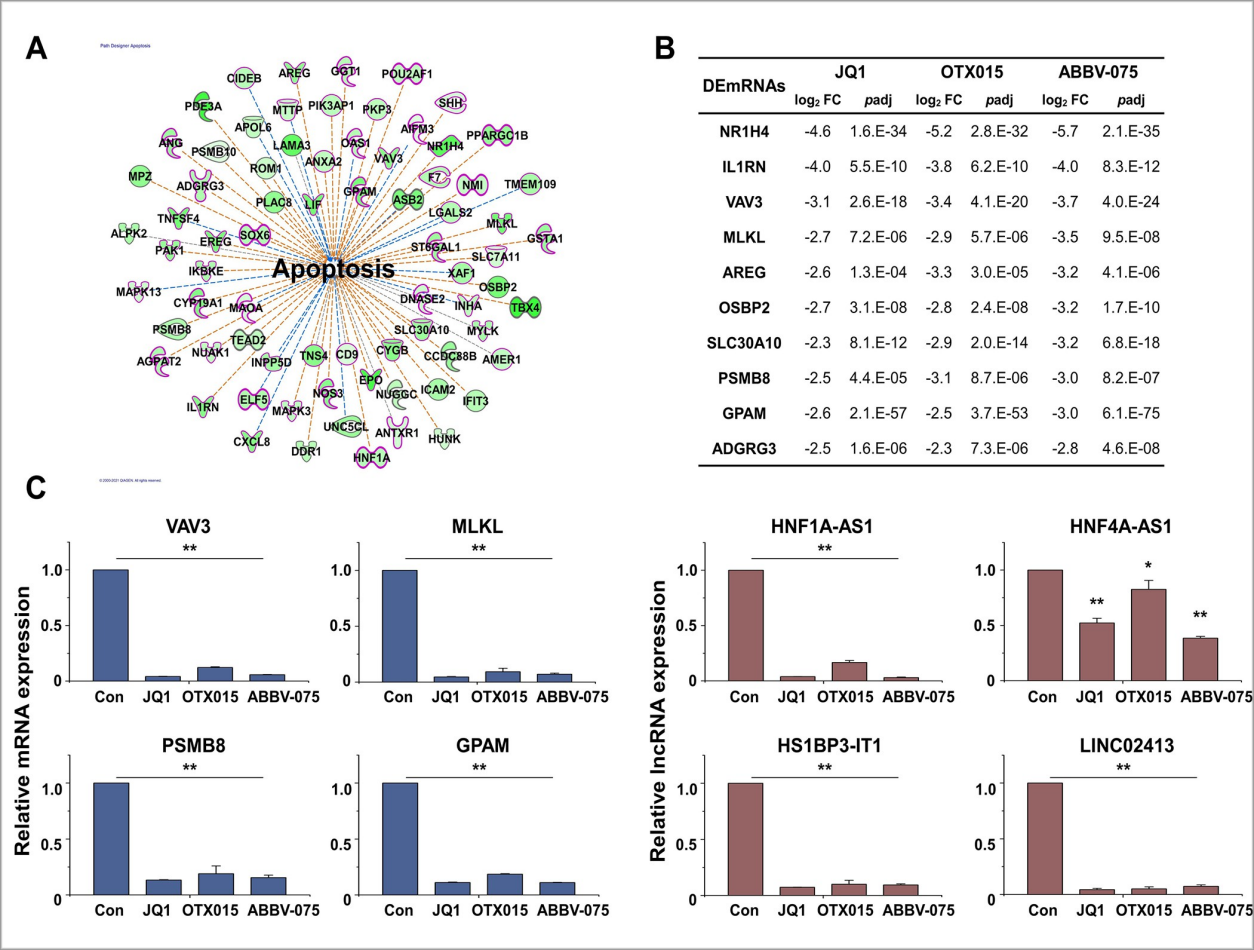
Biofunctional analysis of DEmRNAs related to apoptosis. (A) IPA downstream effects analysis showing the mapping of most significant genes related to apoptosis from the results of the biofunctional analysis in Module 1. Of the genes related to apoptosis, only cell death and survival-related genes are shown in bold and red. (B) Log_2_ fold changes and *p*adj in the expression of genes related to apoptosis. (C) Expression levels of apoptosis-related mRNAs and lncRNAs were analyzed by qRT-PCR and normalized to GAPDH or U6 transcript levels, respectively. Overall, the apoptosis-related mRNAs and lncRNAs were inhibited by BET inhibitors. The data represent three independent experiments. The values are the mean ± SD of triplicate experiments (*p < 0.05 and **p < 0.01).

The apoptosis-related genes of the IPA downstream effect analysis are shown in Fig 6A. Of the genes related to apoptosis, only the top 10 genes related to cell death and survival are listed in Fig 6B. The selected DEmRNAs and DElncRNAs related to apoptosis were validated using qRT-PCR (Fig 6C).

**Fig 7:**
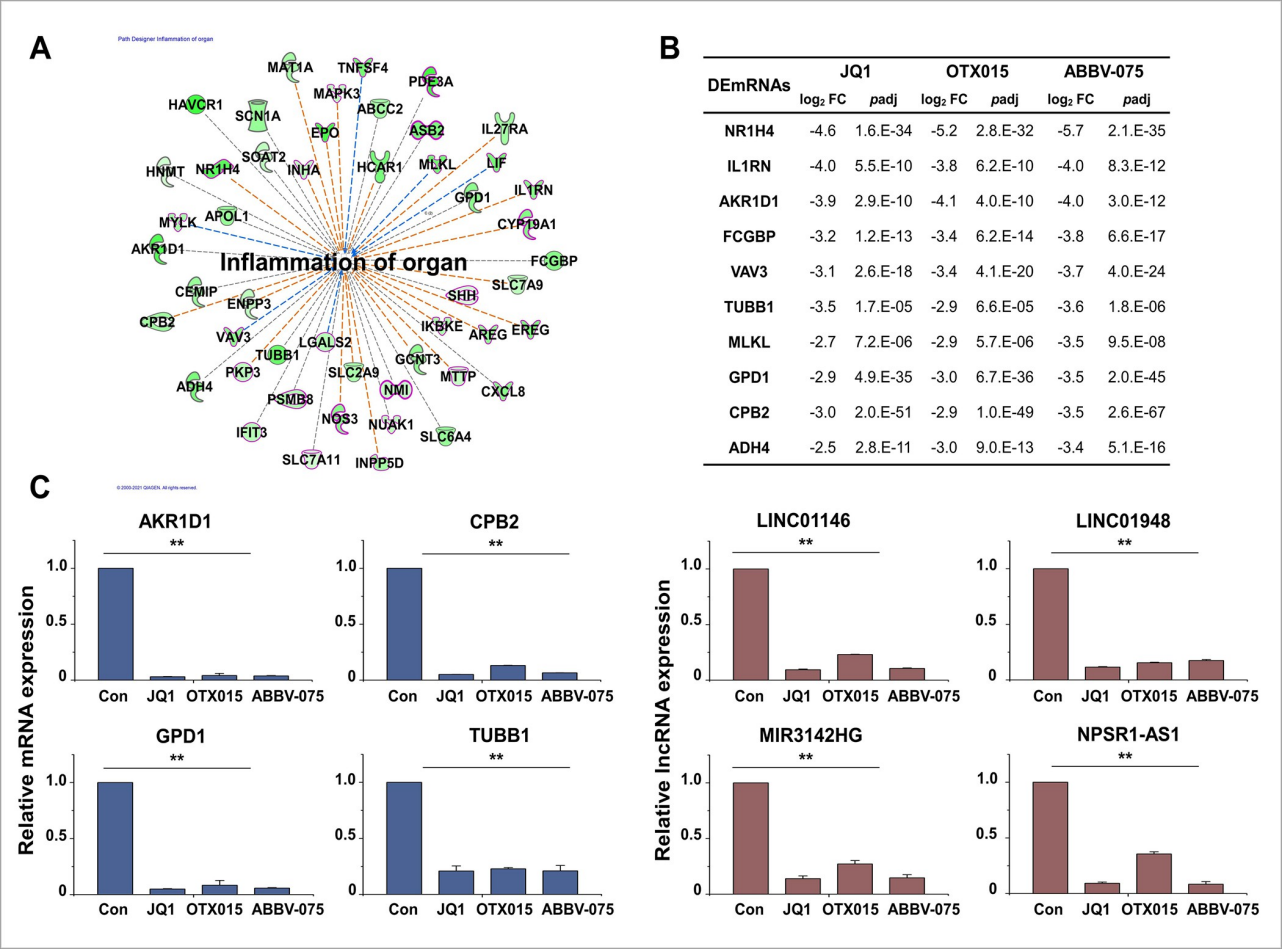
Biofunctional analysis of DEmRNAs related to inflammation of the organ. (A) IPA downstream effects analysis showing the mapping of most significant genes related to organ inflammation from the results of the biofunctional analysis in Module 1. Of the genes related to inflammation of organ, only apoptosis-related genes are shown in bold and red. (B) Log_2_ fold changes and *p*adj in the expression of genes related to inflammation of the organ. (C) Expression levels of inflammation of organ-related mRNAs and lncRNAs were analyzed by qRT-PCR and normalized to GAPDH or U6 transcript levels, respectively. Overall, the inflammation of organ-related mRNAs and lncRNAs was inhibited by BET inhibitors. The data represent three independent experiments. The values are the mean ± SD of triplicate experiments (**p < 0.01).

The inflammation of organ-related genes of the IPA downstream effect analysis is shown in Fig 7A. Of the genes related to the inflammation of the organ, only the top 10 genes related to apoptosis are listed in Fig 7B. The selected DEmRNAs and DElncRNAs related to the inflammation of organs were validated using qRT-PCR (Fig 7C).

**Fig 8:**
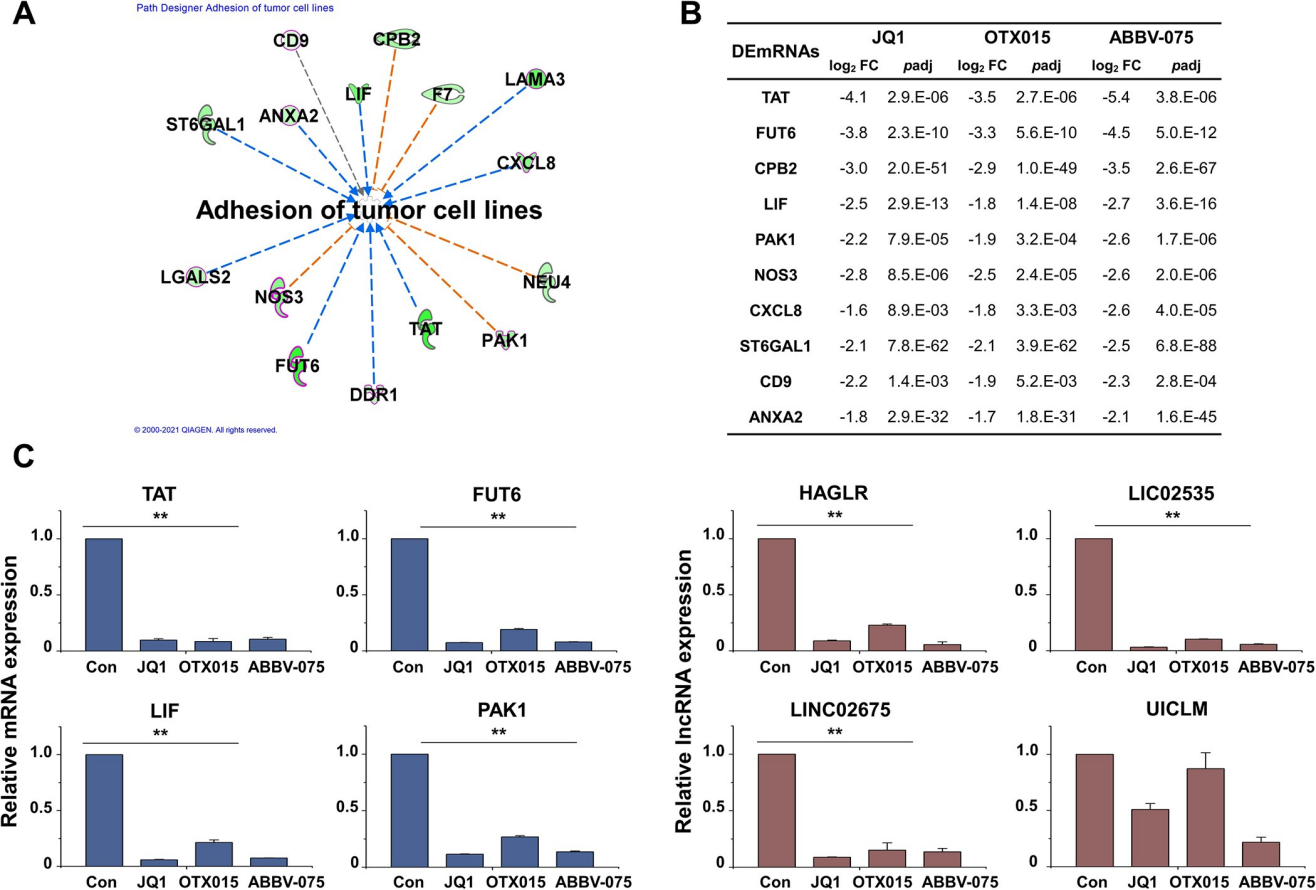
Biofunctional analysis of DEmRNAs related to adhesion of tumor cell lines. (A) IPA downstream effects analysis showing the mapping of most significant genes related to adhesion of tumor cell lines from the results of the biofunctional analysis in Module 1. Of the genes related to adhesion of tumor cell lines, only metastasis-related genes are shown in bold and red. (B) Log_2_ fold changes and *p*adj in the expression of genes related to adhesion of tumor cell lines. (C) Expression levels of adhesion-related mRNAs and lncRNAs were analyzed by qRT-PCR and normalized to GAPDH or U6 transcript levels, respectively. Overall, the adhesion-related mRNAs and lncRNAs were inhibited by BET inhibitors. The data represent three independent experiments. The values are the mean ± SD of triplicate experiments (**p < 0.01).

The adhesion of tumor cell lines-related genes of the IPA downstream effect analysis is shown in Fig 8A. Of the genes related to the adhesion of tumor cell lines, only the top 10 genes related to metastasis are listed in Fig 8B. The selected DEmRNAs and DElncRNAs related to the adhesion of tumor cell lines were validated using qRT-PCR (Fig 8C).

Taken together, we found that most of the DEmRNAs and DElncRNAs commonly found in JQ1, OTX015, and ABBV-075 treatments were downregulated, resulting in decreased proliferation and adhesion and increased apoptosis and inflammation. These data strongly suggest that BET inhibitors highly selectively suppress the expression of mRNAs and lncRNAs involved in HCC development and progression.

### Analysis of Module 2—Functional annotation and pathway enrichment analysis

**Fig 9:**
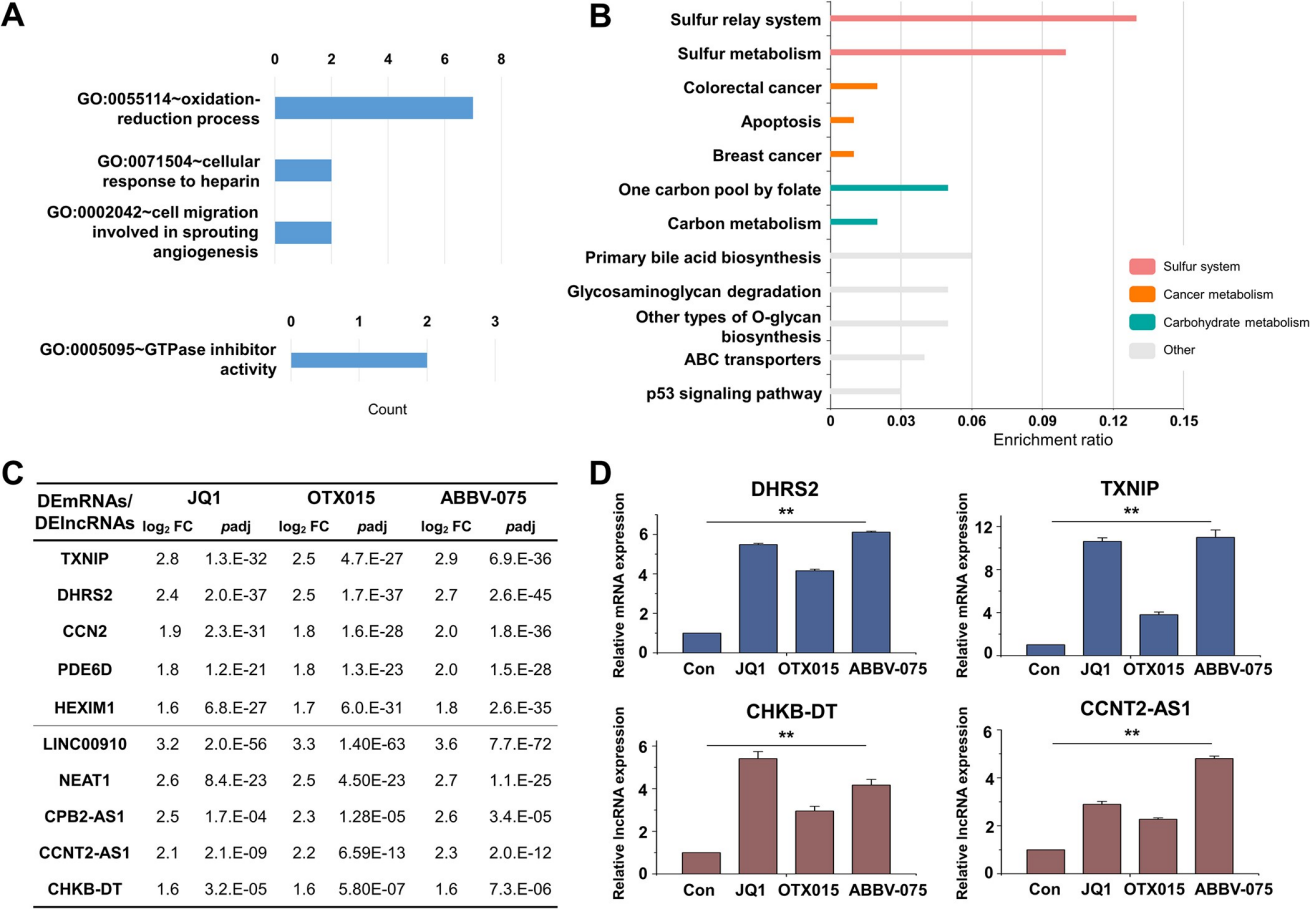
Functional analysis and confirmation of DEmRNAs and DElncRNAs in Module 2. (A) The number of genes enriched for each term are displayed for the GO terms in biological process (upper panel) and molecular function (bottom panel). (B) KEGG pathway enrichment analysis of DEmRNAs. Each row represents an enriched function, and the length of the bar represents the enrichment ratio. (C) Log_2_ fold changes and *p*adj in the expression of genes in Module 2. (D) Expression levels of adhesion-related mRNAs and lncRNAs were analyzed by qRT-PCR and normalized to GAPDH or U6 transcript levels, respectively. Overall, the expressions of adhesion-related mRNAs and lncRNAs were increased by BET inhibitor. The data represent three independent experiments. The values are the mean ± SD of triplicate experiments (**p < 0.01).

Based on the results of the correlation analysis, we performed functional annotation of DEmRNAs of Module 2. The top 5 GO terms of the BP, CC, and MF are shown in Fig 9A. In the BP categories, most enriched GO terms were mainly related to the oxidation-reduction process, cellular response to heparin, and cell migration involved in sprouting angiogenesis. There were no significant results in the CC category. In the MF categories, the only significant term was related to GTPase inhibitor activity. The KEGG pathway results showed sulfur metabolism, primary bile acid biosynthesis, and glycosaminoglycan degradation, suggesting that the most enriched KEGG pathways were mainly involved in metabolic pathways. We also performed GSEA of DEmRNAs of Module 2 (S2C Fig). The GSEA results showed that DEmRNAs of Module 2 were negatively correlated with genes involved in the homeostatic process. The top 5 DEmRNAs and DElncRNAs in Module 2 are listed in Fig 9C. The selected DEmRNAs and DElncRNAs were validated using qRT-PCR (Fig 9D). As shown by RNA-seq, the selected DEmRNAs and DElncRNAs were upregulated by the BET inhibitors.

In addition, we further validated a novel putative biomarker in the human HCC cell line Huh7. Huh7 cells were treated with BET inhibitors under the same conditions as HepG2. The DEmRNAs and DElncRNAs were validated using qRT-PCR. The expressions of DEmRNAs (S5 Fig) and DElncRNAs (S6 Fig) involved in HCC progression, apoptosis, and inflammation were consistent with the results of HepG2.

## Discussion

Epigenetic inhibition with BET inhibitors is emerging as a therapy for a broad range of cancers, including HCC [2, 3]. Here, we conducted a genome-wide analysis of the transcript changes potentially underlying by treating the human HCC cell lines HepG2 and Huh7 with the BET inhibitors JQ1, OTX015, or ABBV-075 the inhibition of HCC progression. Selected DEmRNAs and DElncRNAs identified in this study are highlighted below.

JQ1 and OTX015 are known to inhibit the expression of active enhancer-associated genes, c-MYC oncogene, MYCN transcription factor, and its target genes involved in HCC progression and tumorigenesis [10, 40]. These BET inhibitors also inhibit HCC cell proliferation and migration in vitro [39]. In that study, JQ1 and OTX015 selectively inhibited the expression of cell migration-related genes such as *ASIC1*, *CD9*, *SSTR5*, and *VAV3* through *SMARCA4*. In line with this, we showed here that the BET inhibitors reduced the expression of proliferation- or migration-related genes such as *AREG*, *CD9*, *EREG*, and *VAV3*. *VAV3* (Vav guanine nucleotide exchange factor 3) counteracts apoptosis and is overexpressed in various cancers [41, 42]. ABBV-075 showed anti-apoptotic activity in small-cell lung cancer [43] and non-Hodgkin lymphoma cell lines [44], at least in the latter with a more significant effect than OTX015. Similarly, ABBV-075 was more efficacious than OTX015 in the HepG2 cells. However, the expression levels in the Huh7 cells were not significantly different between each group. OTX015 remains of interest since it showed anticancer effects in many studies and is currently in clinical trials.

The present study also identified cancer-relevant DEmRNAs that have not previously been associated with HCC. Amongst the novel DEmRNAs that JQ1, OTX015, and ABBV-075 commonly downregulated are transcripts such as *ADGPR3*, *GPAM*, *GPD1*, *OSBP2*, *PSMB8*, *SLC6A9*, and *SLC30A10*. The expression of *GPAM* (glycerol-3-phosphate acyltransferase), a key enzyme in lipid biosynthesis, is increased in breast and other cancers and highly correlated with clinicopathological parameters [45]; its inhibition reduces ovarian cancer cell migration and the growth of tumor xenograft mouse models [46]. A high expression of *GPD1* (glycerol-3-phosphate dehydrogenase 1) has been observed in glioblastoma and glioma and correlates with poor survival in renal cell carcinoma [47]. The expression of *PSMB8* (proteasome subunit beta type 8), a component of the 20S proteolytic core particle, is generally increased in tumor cell lines and correlated with poor prognosis [48]; its reduction inhibits the progression and invasion of gastric cancer and glioblastoma [49].

We also found that DEmRNAs involved in cancer growth and progression were upregulated by the three BET inhibitors. For example, *HEXIM1* has previously been described as a pharmacodynamic marker in BET treatments [50]. Noteworthy novel DEmRNAs are *CNNM4* and *USP53*. *CNNM4* is a member of the cyclin and cystathionine β-synthase (CBS) domain divalent metal cation transport mediator family and promotes tumor progression by regulating Mg^2+^ efflux; its level is inversely correlated with malignancy in colon cancer [51]. *USP53* (Ubiquitin specific peptidase 53) inhibits the initiation and progression of renal cell carcinoma by inhibiting activation of the nuclear factor κB (*NF‐κB*) pathway; it promotes apoptosis and inhibits glycolysis in lung adenocarcinoma through FKBP51‐AKT1 signaling [52, 53].

Recent evidence suggests that lncRNAs are key modulators of HCC by regulating tumor suppressor genes or oncogenes [22–26, 54]. Here, we found 60 DElncRNAs in BET inhibitor-treated HepG2 cells. Some of the downregulated DElncRNAs have previously been associated with HCC. For example, *HAGLR*, *HNF1A-AS1*, and *PART1* are known to be upregulated in HCC and to promote HCC proliferation and migration [55–57], while the downregulation of *LINC00261* is inconsistent with a previous study [58]. Some of the downregulated DElncRNAs that our study newly associates with HCC have been related to other cancers and pathologies. Thus, *LINC00242* and *LINC02535* affect the proliferation and apoptosis of gastric cancer [59, 60], *PRR7-AS1*is involved in colorectal cancer metabolism [61], and *LINC01146* and *MIR3142HG* in inflammation [62, 63]. Noteworthy upregulated novel DElncRNAs in the BET inhibitor-treated HepG2 cells include *CCNT2-AS1*, *CHKB-DT*, *CPB2-AS1*, and *LINC00910*. Of these, *CCNT2-AS1* has a prognostic value in renal cell carcinoma [64], and *CHKB-DT*, also known as *CHKB-AS1* has been negatively correlated with esophageal squamous cell carcinoma [65]. In this study, DElncRNAs and DEmRNAs were paired with the absolute value of the Pearson correlation coefficient. Further studies will be needed to demonstrate the function of novel DElncRNAs in HCC, their interactions with target genes, and the specific lncRNA-mRNA pairs involved in HCC progression.

Several BET inhibitors, including ABBV-075, CPI-0610, GSK525762, and OTX015, are currently in phase 1 or 2 clinical trials [13, 15]. However, the pan-BET inhibitors, especially the oldest such as JQ1, have limited clinical applications due to their toxicity, side effects, and short clinical response [66]. Recently, selective inhibitors targeting BD1 and BD2 have been developed. The BD1 inhibitor, GSK078, showed efficacy in cancer models comparable to the pan-BET inhibitor. The BD2 inhibitors, GSK046 and ABBV-744, were predominantly effective in inflammatory and autoimmune disease models [67, 68]. The present study will inform future efforts to optimize further the development and application of the different BET inhibitors for the treatment of HCC.

## Conclusions

We performed a comprehensive analysis of DEmRNAs and DElncRNAs expression profiles and their functional annotation in the BET inhibitor-treated human HCC cell line HepG2. We identified 382 DEmRNAs and 60 DElncRNAs in common for the BET inhibitors, JQ1, OTX015, and ABBV-075, and analyzed the correlation between DEmRNAs and DElncRNAs based on their expression profiles. Additionally, we found novel transcripts involved in proliferation and apoptosis that are commonly reduced by the three inhibitors. Novel DEmRNAs are *ADGPR3*, *GPAM*, *GPD1*, *OSBP2*, *PSMB8*, *SLC6A9*, *SLC30A10*, and novel DElncRNAs are *LINC00242*, *LINC01146*, *LINC02535*, *MIR3142HG*, and *PRR7-AS1*. Our study suggests that alterations at the level of epigenetic components may modulate HCC progression, providing biomarkers for therapeutic targeting.

## Supporting information

S1 Fig Effects of BET inhibitor on HCC cell line HepG2. HepG2 cells were treated with JQ1, OTX015, or ABBV-075 at different concentrations for different durations. The viability of the HepG2 cells was determined using the WST-1 assay. The data represent three independent experiments. The values are the mean ± SD of triplicate experiments (*p < 0.05 and **p < 0.01).
(TIF)

S2 Fig Gene set enrichment analysis (GSEA) of DEmRNAs in each module. The degree of enrichment of total DEmRNAs (A), DEmRNAs in Module 1 (B), and Module 2 (C) represents a normalized enrichment score (NES). The right panel of each graph shows the GSEA enrichment plot.
(TIF)

S3 Fig Functional annotation of commonly expressed lncRNAs treated with three BET inhibitors. GREAT computes all GO term enrichment for genes upstream and downstream of TSS based on the genomic regions of DElncRNAs commonly expressed in the three BET inhibitor treatments. (A) The number of associated genes in *cis*-regulatory genomic regions. (B) Distance (kb) to the nearest transcriptional start site (TSS) of commonly expressed DElncRNAs in the three BET inhibitor treatments. (C) Absolute distance to DElncRNAs and TSS sites. (D) Functional annotation analysis of DElncRNAs using GREAT shows for the top 5 GO terms in BP (top panel), CC (middle panel), and MF (bottom panel).
(TIF)

S4 Fig Functional annotation and pathway enrichment analysis of DEmRNAs of each BET inhibitor treatment. HepG2 cells were treated with JQ1 (A), OTX-015 (B), and ABBV-075 (C). The top 5 enriched GO terms (top panel), KEGG pathways (middle panel), and GSEA (bottom panel) show the results of functional analysis of up- and down-regulated DEmRNAs. Different colors represent up- (red) and downregulated (blue) DEmRNAs.
(TIF)

S5 Fig Verification of DEmRNAs in Human HCC cell line Huh7. Expression levels of DEmRNAs in Huh7 cells were analyzed by qRT-PCR and normalized to GAPDH transcript levels. The data represent three independent experiments. The values are the mean ± SD of triplicate experiments (**p < 0.01).
(TIF)

S6 Fig Verification of DElncRNAs in Human HCC cell line Huh7. Expression levels of DElncRNAs in Huh7 cells were analyzed by qRT-PCR and normalized to U6 transcript levels. The data represent three independent experiments. The values are the mean ± SD of triplicate experiments (**p < 0.01).
(TIF)

S1 Table List of primers for qRT-PCR. (DOCX)

S2 Table Top 50 significant up- and downregulated DEmRNAs in JQ1-treated HepG2 cells. (DOCX)

S3 Table Top 50 significant up- and downregulated DElncRNAs in JQ1-treated HepG2 cells. (DOCX)

S4 Table Top 50 significant up- and downregulated DEmRNAs in OTX015-treated HepG2 cells. (DOCX)

S5 Table Top 50 significant up- and downregulated DElncRNAs in OTX015-treated HepG2 cells. (DOCX)

S6 Table Top 50 significant up- and downregulated DEmRNAs in ABBV-075-treated HepG2 cells. (DOCX)

S7 Table Top 50 significant up- and downregulated DElncRNAs in ABBV-075-treated HepG2 cells. (DOCX)

S8 Table Top 50 significant up- and downregulated DEmRNAs in BET inhibitor-treated HepG2 cells in common. (DOCX)

S9 Table Top 50 significant up- and downregulated DElncRNAs in BET inhibitor-treated HepG2 cells in common. (DOCX)

S10 Table Pearson correlation coefficients (PCC) value of DElncRNA-DEmRNA pairs in Module 1. (XLSX)

S11 Table Pearson correlation coefficients (PCC) value of DElncRNA-DEmRNA pairs in Module 2. (XLSX)

## Abbreviations

BET: Bromodomain and extra-terminal
BP: Biological process
BRD: Bromodomain-containing protein
CC: Cellular component
DAVID: Database for annotation, visualization and integrated discovery
DEmRNA: Differentially expressed mRNAs
DElncRNA: Differentially expressed lncRNAs
GO: Gene ontology
GREAT: Genomic regions enrichment of annotations tool
HCC: Hepatocellular carcinoma
IPA: Ingenuity pathway analysis
KEGG: Kyoto encyclopedia of genes and genomes
LncRNA: Long non-coding RNAs
MF: Molecular function
PCC: Pearson correlation coefficient
qRT-PCR: Quantitative reverse transcription-polymerase chain reaction
RNA-seq: RNA sequencing
