# Supplementary figures and images for: Comprehensive transcriptome profiling of BET inhibitor-treated HepG2 cells
Source: PLoS One. 2022 Apr 29;17(4):e0266966. doi: 10.1371/journal.pone.0266966 (PMC9053788; doi:10.1371/journal.pone.0266966)

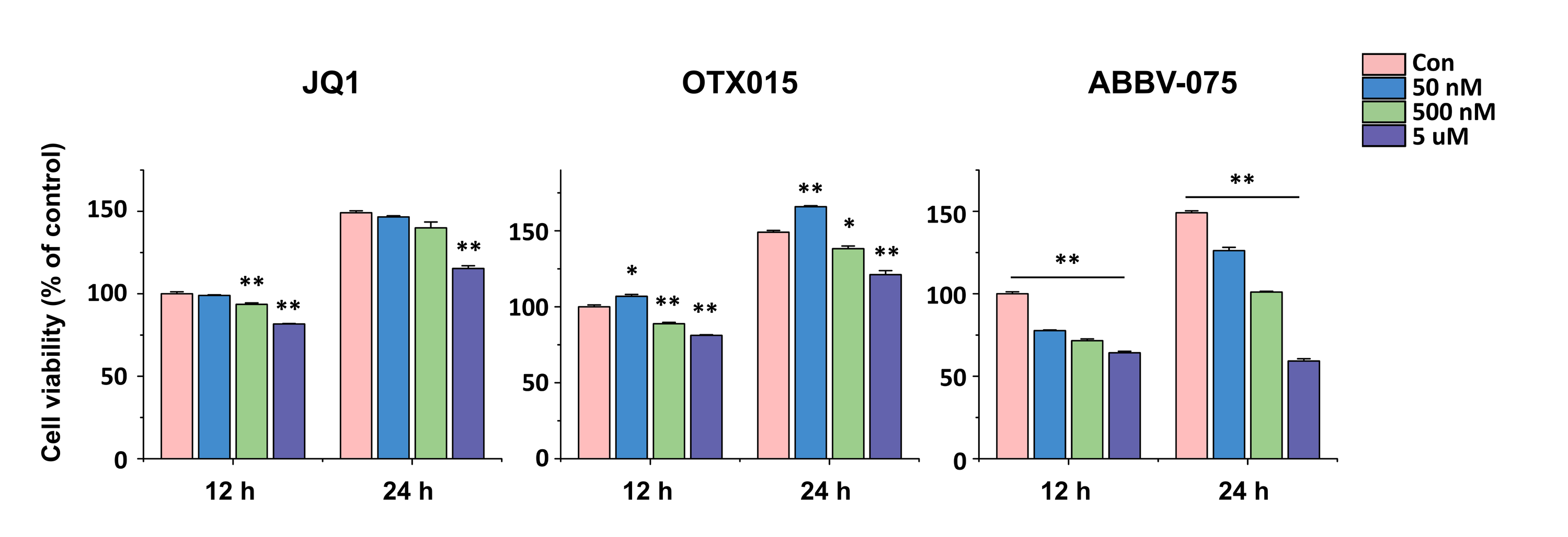

Supplement: S1 Fig — HepG2 cells were treated with JQ1, OTX015, or ABBV-075 at different concentrations for different durations. The viability of the HepG2 cells was determined using the WST-1 assay. The data represent three independent experiments. The values are the mean ± SD of triplicate experiments (*p < 0.05 and **p < 0.01). (TIF) [file pone.0266966.s001.tif]

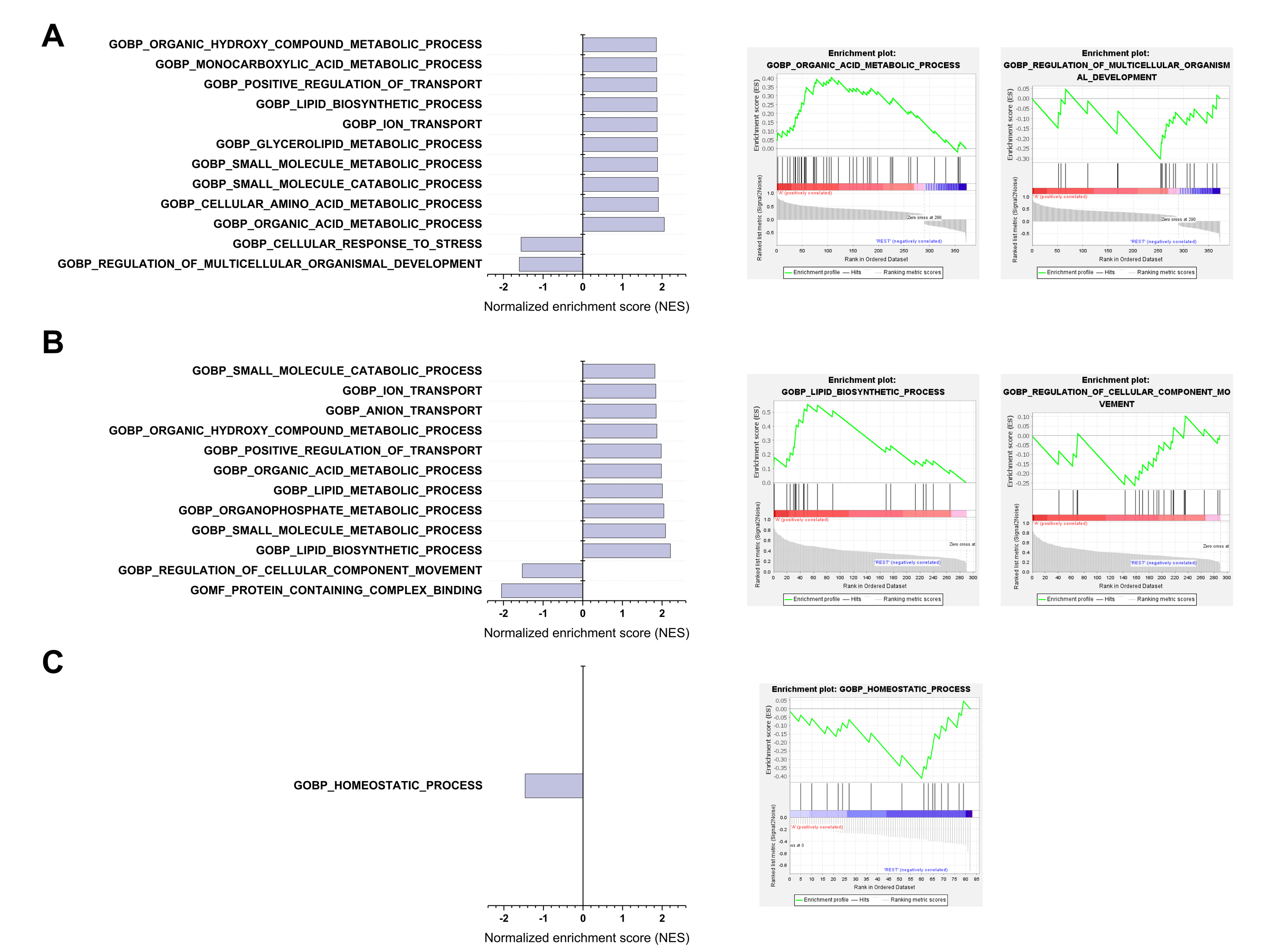

Supplement: S2 Fig — The degree of enrichment of total DEmRNAs (A), DEmRNAs in Module 1 (B), and Module 2 (C) represents a normalized enrichment score (NES). The right panel of each graph shows the GSEA enrichment plot. (TIF) [file pone.0266966.s002.tif]

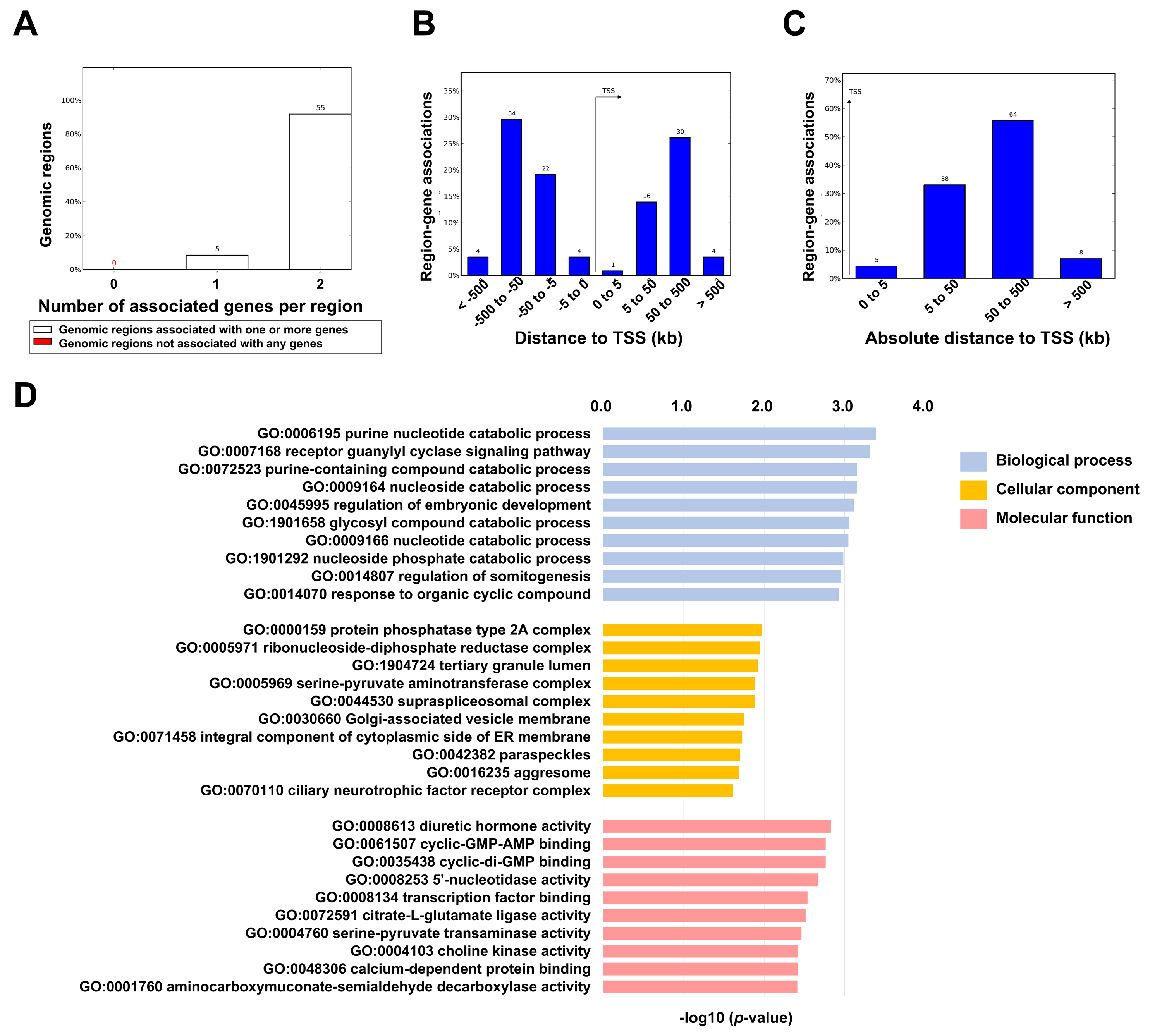

Supplement: S3 Fig — GREAT computes all GO term enrichment for genes upstream and downstream of TSS based on the genomic regions of DElncRNAs commonly expressed in the three BET inhibitor treatments. (A) The number of associated genes in cis-regulatory genomic regions. (B) Distance (kb) to the nearest transcriptional start site (TSS) of commonly expressed DElncRNAs in the three BET inhibitor treatments. (C) Absolute distance to DElncRNAs and TSS sites. (D) Functional annotation analysis of DElncRNAs using GREAT shows for the top 5 GO terms in BP (top panel), CC (middle panel), and MF (bottom panel). (TIF) [file pone.0266966.s003.tif]

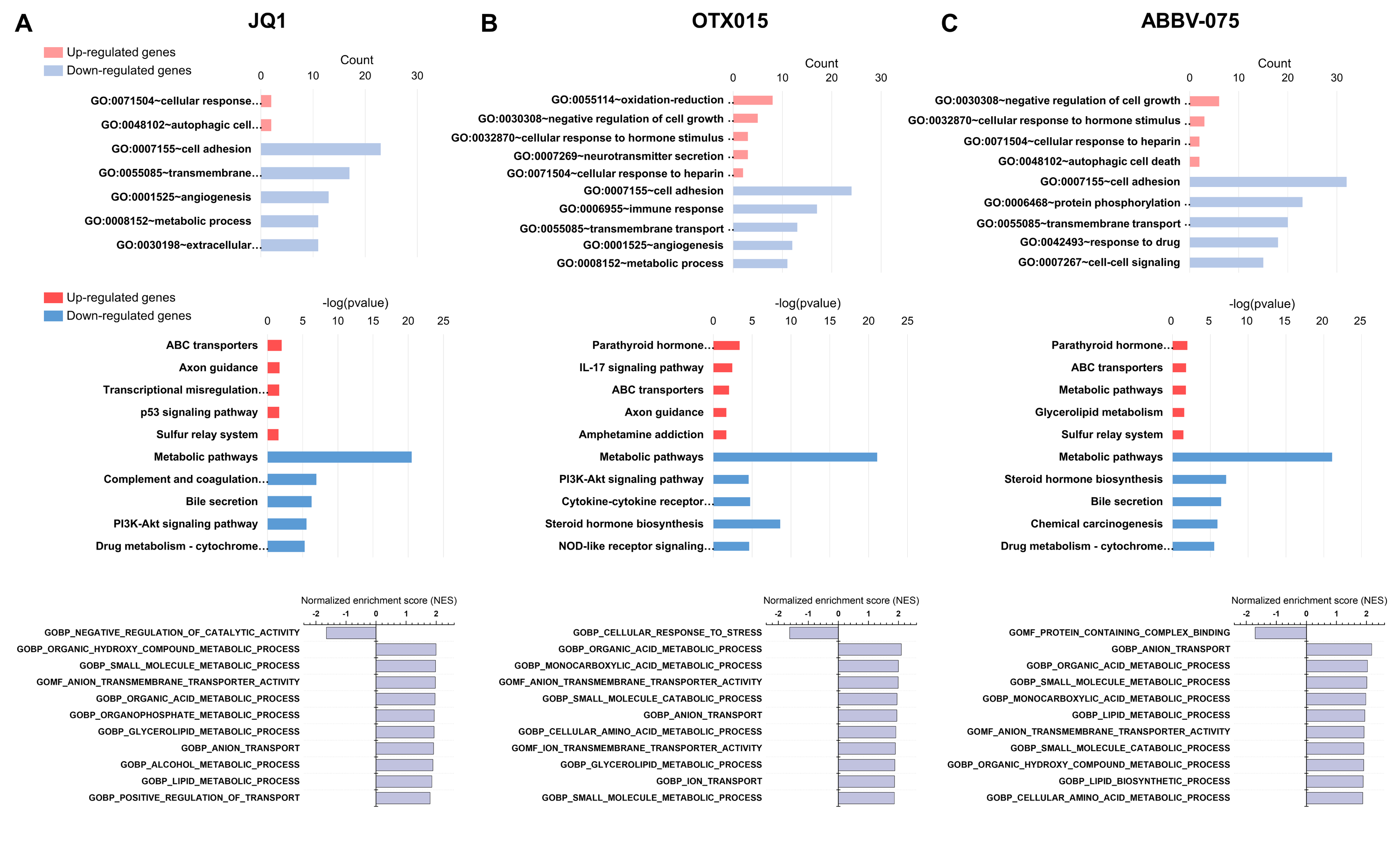

Supplement: S4 Fig — HepG2 cells were treated with JQ1 (A), OTX-015 (B), and ABBV-075 (C). The top 5 enriched GO terms (top panel), KEGG pathways (middle panel), and GSEA (bottom panel) show the results of functional analysis of up- and down-regulated DEmRNAs. Different colors represent up- (red) and downregulated (blue) DEmRNAs. (TIF) [file pone.0266966.s004.tif]

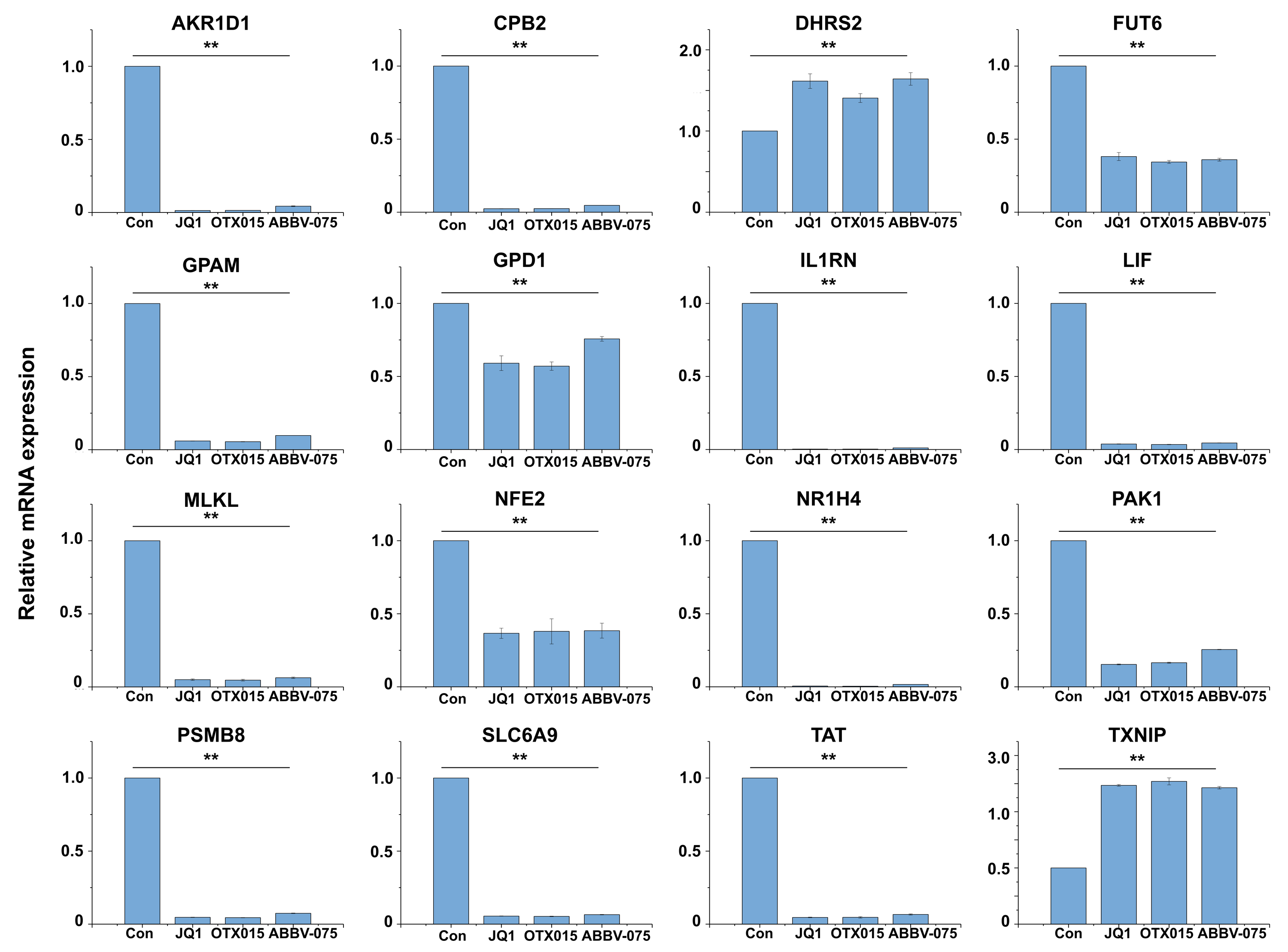

Supplement: S5 Fig — Expression levels of DEmRNAs in Huh7 cells were analyzed by qRT-PCR and normalized to GAPDH transcript levels. The data represent three independent experiments. The values are the mean ± SD of triplicate experiments (**p < 0.01). (TIF) [file pone.0266966.s005.tif]

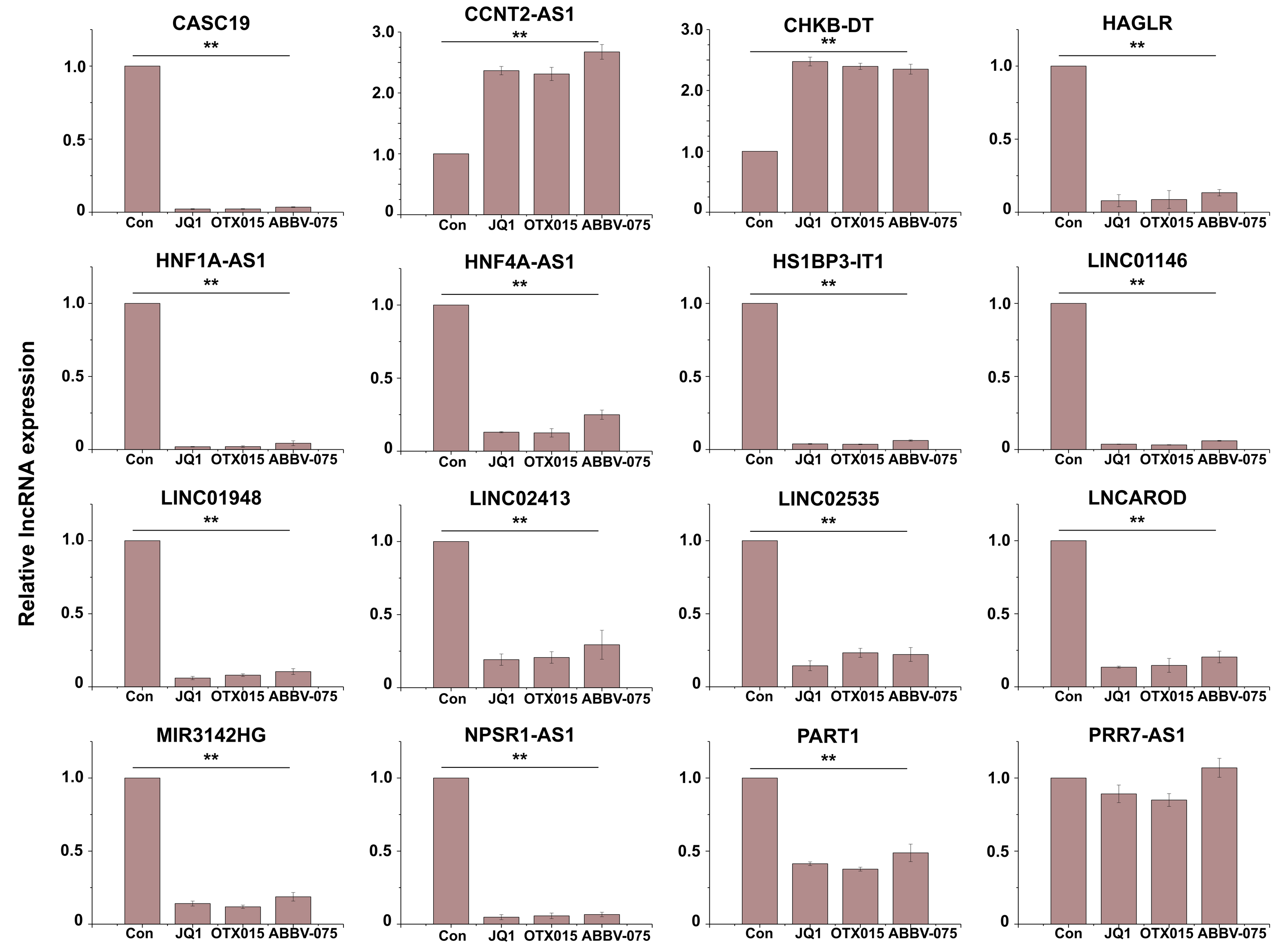

Supplement: S6 Fig — Expression levels of DElncRNAs in Huh7 cells were analyzed by qRT-PCR and normalized to U6 transcript levels. The data represent three independent experiments. The values are the mean ± SD of triplicate experiments (**p < 0.01). (TIF) [file pone.0266966.s006.tif]
