# Supplementary material for: Comprehensive transcriptome profiling of BET inhibitor-treated HepG2 cells
Source: PLoS One. 2022 Apr 29;17(4):e0266966. doi: 10.1371/journal.pone.0266966 (PMC9053788; doi:10.1371/journal.pone.0266966)
Supplement: S1 Table — (DOCX) [file pone.0266966.s007.docx]

**S1 Table. List of primers for qRT-PCR.**

| **mRNAs**  **/lncRNAs** | **Forward (5′ → 3′)** | **Reverse (5′ → 3′)** |
| --- | --- | --- |
| AKR1D1 | TCAGAACCTAAATCGACCCCT | TCCCCAACTTCGTGTTCATTTT |
| CPB2 | GAACTGTCTCTAGTAGCCAGTGA | TGCCCAAATCATAGATCCAATCG |
| DHRS2 | AGGCACCGAGTATTTTGTGCT | CCTGCTGGATAGAACTTGCCAT |
| FUT6 | CCG ACT ACA TCA CCG AGA AGC T | GAA CCT CTC GTA GTT GCT TCT GC |
| GPAM | GATGTAAGCACACAAGTGAGGA | TCCGACTCATTAGGCTTTCTTTC |
| GPD1 | GCCATCTGAAGGCAAACGC | GCCAATGGTTGTCTCACAGAAC |
| IL1RN | GACCTTCTATCTGAGGAACAACC | CTCAATGGGTACCACATCTATCTT |
| LIF | CCAACGTGACGGACTTCCC | TACACGACTATGCGGTACAGC |
| MLKL | AGGAGGCTAATGGGGAGATAGA | TGGCTTGCTGTTAGAAACCTG |
| NFE2 | GCAGGAACAGGGTGATACAGC | GCAGCTCGGTGATGGACAT |
| NR1H4 | GACTTTGGACCATGAAGACCAG | GCCCAGACGGAAGTTTCTTATT |
| PAK1 | CAACTCGGGACGTGGCTAC | CAGTATTCCGGGTCAAAGCAT |
| PSMB8 | GGTCCTACATTAGTGCCTTACGG | CGCAGATAGTACAGCCTGCATT |
| SLC6A9 | CAGATCGAGTTTGTACTGACGAG | GCGATAGCAGAGGTATGGGAAG |
| TAT | CTGGACTCGGGCAAATATAATGG | GTCCTTAGCTTCTAGGGGTGC |
| TUBB1 | CTACAACGCGGTTCTGTCTATC | GGTGGGTGTCGTCAGCTTC |
| TXNIP | TGTGTGAAGTTACTCGTGTCAAA | GCAGGTACTCCGAAGTCTGT |
| VAV3 | AGAGAAACGGACCAATGGACT | GGTGGTGTTCCAGAATAGTTCC |
| CASC19 | TGCCATACAGTGGTTTTTGGG | TGGGGCATGACACCCTTTTG |
| CCNT2-AS1 | TTTACGGATGAGGGACCACG | GCTCCCTTGGCTTGTAGGTT |
| CHKB-DT | GGCTACCCCTGTGCTTGAAC | CTGAGGCGATGTGATTCGTG |
| HAGLR | GGGCTGGTACAGACTAGGGA | TAAGCAGGTCAGAAAGGGCG |
| HNF1A-AS1 | TTGCTTTTTCAGAGAGCCAG | AATGCGAGGTGTTTCAGGTA |
| HNF4A-AS1 | TCCTTTAGCGGCTTCCAAGG | GCCTGTCTTCAAGTCACCGA |
| HS1BP3-IT1 | CTGGGCTTACTCCTAGCTGC | CTTGTGTTCTTGGGTGCTGC |
| LINC00242 | TTCAGGCGCTGTCTGTTCTT | TGCGAATCGATGGGGATCAG |
| LINC01146 | AGCGTTTTTGTTTGCACATCCT | ACCTGGACTGCTTTGCTGAT |
| LINC01948 | TTGGAGCCAAGGCAGACAAA | ATTCCAGCTCAGCCGAAACA |
| LINC02413 | GCACACAATACACCGAGCAAG | CCTCTTGATGACTCTGGGCTC |
| LINC02535 | AAGGAGCTCTGTTCTCCAGG | GCCTCTATGTAGGGCGCTTT |
| LINC02348 | CAAGTGTGGGAATGGCCTCC | TGCTCACATCTCTGGGGAGT |
| LINC02675 | AAGGCAGTCAAGGTGTGGAG | AAGGGTGAAGTGCCAATCCT |
| LNCAROD | CCACAACGGCAACCAGTAAAA | TAGGCGTTCCACCTGCAAAT |
| MIR3142HG | GAGGGGTCTTTGCACCATCT | CAAGCCCACGATGACAGAGA |
| NPSR1-AS1 | TGTTGAGAAGTGCACGGTCC | GGCATGTGGTGACTATGCCA |
| PART1 | GGACTCGTGCTTCTCGTACGCTGG | GCCTGCCCTTTGGTTTCTGGGAC |
| PRR7-AS1 | GGGGCACTTGTGATCCAGAC | CTCTGCTCCGTGTTCCTAGC |
| TUSC8 | TGTGAAGAGGAGAACATAAAAGG | AAGCAAGATAATACAGTGGCGA |
| UICLM | GGATGAGGAGACATCCACGG | GAGTCCTGACTGGCGTTGAA |
