# Supplementary material for: Comprehensive transcriptome profiling of BET inhibitor-treated HepG2 cells
Source: PLoS One. 2022 Apr 29;17(4):e0266966. doi: 10.1371/journal.pone.0266966 (PMC9053788; doi:10.1371/journal.pone.0266966)
Supplement: S2 Table — (DOCX) [file pone.0266966.s008.docx]

**S2 Table. Top 50 significant up- and downregulated DEmRNAs in JQ1-treated HepG2 cells.**

| **Ensembl_id** | **mRNA_symbol** | **Log2FoldChange** | ***p*adj** |
| --- | --- | --- | --- |
| ENSG00000113657.13 | DPYSL3 | 6.1 | 2.9.E-10 |
| ENSG00000084710.14 | EFR3B | 5.1 | 2.7.E-52 |
| ENSG00000103056.12 | SMPD3 | 4.7 | 2.9.E-04 |
| ENSG00000116991.11 | SIPA1L2 | 4.7 | 1.7.E-03 |
| ENSG00000010030.14 | ETV7 | 4.4 | 1.1.E-02 |
| ENSG00000103196.12 | CRISPLD2 | 4.2 | 1.3.E-03 |
| ENSG00000145147.20 | SLIT2 | 3.9 | 1.1.E-03 |
| ENSG00000166750.10 | SLFN5 | 3.9 | 5.5.E-03 |
| ENSG00000232466.1 | H3P31 | 3.8 | 1.9.E-02 |
| ENSG00000227766.1 | AL671277.1 | 3.8 | 3.2.E-02 |
| ENSG00000197380.11 | DACT3 | 3.8 | 2.2.E-02 |
| ENSG00000149260.18 | CAPN5 | 3.8 | 5.5.E-09 |
| ENSG00000254550.1 | OMP | 3.7 | 2.7.E-02 |
| ENSG00000163995.21 | ABLIM2 | 3.7 | 1.3.E-02 |
| ENSG00000283676.1 | MIR5087 | 3.7 | 1.5.E-02 |
| ENSG00000206848.1 | RNU6-890P | 3.7 | 3.7.E-02 |
| ENSG00000165807.8 | PPP1R36 | 3.6 | 2.7.E-02 |
| ENSG00000163220.11 | S100A9 | 3.6 | 9.2.E-03 |
| ENSG00000214456.8 | PLIN5 | 3.5 | 6.1.E-09 |
| ENSG00000170629.14 | DPY19L2P2 | 3.5 | 3.5.E-03 |
| ENSG00000095587.9 | TLL2 | 3.4 | 1.9.E-02 |
| ENSG00000280407.2 | AC132872.4 | 3.4 | 2.4.E-02 |
| ENSG00000196376.11 | SLC35F1 | 3.4 | 2.9.E-02 |
| ENSG00000226632.1 | UBE2V1P1 | 3.4 | 3.5.E-02 |
| ENSG00000224672.4 | RPL17P10 | 3.3 | 4.4.E-02 |
| ENSG00000229992.1 | HMGB3P9 | 3.2 | 4.9.E-02 |
| ENSG00000183793.14 | NPIPA5 | 3.2 | 2.9.E-02 |
| ENSG00000173805.16 | HAP1 | 3.1 | 3.6.E-02 |
| ENSG00000242960.1 | FTH1P23 | 3.1 | 4.3.E-02 |
| ENSG00000145198.14 | VWA5B2 | 3 | 1.3.E-03 |
| ENSG00000144369.13 | FAM171B | 3 | 8.6.E-03 |
| ENSG00000278952.1 | AP003068.4 | 3 | 4.9.E-02 |
| ENSG00000120329.7 | SLC25A2 | 2.9 | 3.9.E-02 |
| ENSG00000100027.17 | YPEL1 | 2.9 | 7.0.E-04 |
| ENSG00000241120.1 | HMGN1P8 | 2.8 | 4.1.E-02 |
| ENSG00000166963.13 | MAP1A | 2.8 | 1.4.E-02 |
| ENSG00000135709.12 | KIAA0513 | 2.8 | 1.3.E-09 |
| ENSG00000166323.13 | C11orf65 | 2.8 | 1.4.E-02 |
| ENSG00000183508.5 | TENT5C | 2.8 | 4.5.E-02 |
| ENSG00000265972.6 | TXNIP | 2.8 | 1.3.E-32 |
| ENSG00000127415.13 | IDUA | 2.7 | 5.9.E-05 |
| ENSG00000223203.1 | RNA5SP221 | 2.7 | 4.5.E-06 |
| ENSG00000280278.1 | FLJ30679 | 2.7 | 6.6.E-03 |
| ENSG00000047936.11 | ROS1 | 2.6 | 3.5.E-02 |
| ENSG00000232859.10 | LYRM9 | 2.6 | 7.9.E-03 |
| ENSG00000238825.1 | RNVU1-2 | 2.6 | 4.7.E-05 |
| ENSG00000206828.1 | RNVU1-30 | 2.6 | 1.3.E-04 |
| ENSG00000100867.15 | DHRS2 | 2.4 | 2.0.E-37 |
| ENSG00000125266.8 | EFNB2 | 2.4 | 4.0.E-02 |
| ENSG00000103184.12 | SEC14L5 | 2.4 | 2.3.E-02 |
| ENSG00000112337.11 | SLC17A2 | -7 | 2.3.E-07 |
| ENSG00000157131.11 | C8A | -6.6 | 2.2.E-06 |
| ENSG00000112494.10 | UNC93A | -6.3 | 5.1.E-06 |
| ENSG00000123405.14 | NFE2 | -6.2 | 4.8.E-06 |
| ENSG00000146755.11 | TRIM50 | -6.2 | 6.9.E-06 |
| ENSG00000100079.7 | LGALS2 | -6.1 | 1.6.E-05 |
| ENSG00000125895.5 | TMEM74B | -5.9 | 2.8.E-05 |
| ENSG00000167656.5 | LY6D | -5.7 | 1.7.E-04 |
| ENSG00000149527.18 | PLCH2 | -5.7 | 8.6.E-05 |
| ENSG00000117472.10 | TSPAN1 | -5.6 | 2.5.E-04 |
| ENSG00000113249.13 | HAVCR1 | -5.6 | 1.4.E-04 |
| ENSG00000161653.11 | NAGS | -5.6 | 1.1.E-04 |
| ENSG00000161791.14 | FMNL3 | -5.4 | 3.5.E-04 |
| ENSG00000143127.13 | ITGA10 | -5.3 | 3.2.E-04 |
| ENSG00000121075.11 | TBX4 | -5.3 | 7.4.E-04 |
| ENSG00000172478.18 | MAB21L4 | -5.3 | 6.7.E-04 |
| ENSG00000102886.15 | GDPD3 | -5.2 | 3.1.E-08 |
| ENSG00000135074.16 | ADAM19 | -5.2 | 5.1.E-04 |
| ENSG00000132274.16 | TRIM22 | -5.2 | 7.5.E-04 |
| ENSG00000173212.4 | MAB21L3 | -5.1 | 1.1.E-03 |
| ENSG00000165376.12 | CLDN2 | -5.1 | 3.1.E-05 |
| ENSG00000154016.14 | GRAP | -5.1 | 1.9.E-03 |
| ENSG00000158315.11 | RHBDL2 | -5 | 7.3.E-04 |
| ENSG00000102230.14 | PCYT1B | -5 | 1.1.E-21 |
| ENSG00000110777.12 | POU2AF1 | -5 | 1.4.E-03 |
| ENSG00000183145.9 | RIPPLY3 | -5 | 1.3.E-03 |
| ENSG00000167741.11 | GGT6 | -5 | 2.4.E-03 |
| ENSG00000159625.15 | DRC7 | -5 | 1.4.E-03 |
| ENSG00000118514.14 | ALDH8A1 | -5 | 8.0.E-05 |
| ENSG00000205918.9 | PDPK2P | -4.9 | 3.2.E-03 |
| ENSG00000167779.9 | IGFBP6 | -4.9 | 1.6.E-03 |
| ENSG00000100031.19 | GGT1 | -4.9 | 7.6.E-04 |
| ENSG00000073150.14 | PANX2 | -4.9 | 3.0.E-03 |
| ENSG00000248763.2 | AC111000.2 | -4.9 | 5.9.E-03 |
| ENSG00000183773.15 | AIFM3 | -4.8 | 6.8.E-05 |
| ENSG00000141574.8 | SECTM1 | -4.8 | 1.2.E-03 |
| ENSG00000140368.13 | PSTPIP1 | -4.8 | 1.7.E-03 |
| ENSG00000130427.3 | EPO | -4.8 | 2.2.E-03 |
| ENSG00000132965.10 | ALOX5AP | -4.8 | 5.3.E-03 |
| ENSG00000055957.11 | ITIH1 | -4.7 | 3.0.E-03 |
| ENSG00000260105.6 | AOC4P | -4.7 | 5.2.E-03 |
| ENSG00000130701.4 | RBBP8NL | -4.7 | 5.2.E-03 |
| ENSG00000283162.1 | AL390726.5 | -4.7 | 3.8.E-03 |
| ENSG00000012504.15 | NR1H4 | -4.6 | 1.6.E-34 |
| ENSG00000147509.14 | RGS20 | -4.6 | 3.0.E-03 |
| ENSG00000262904.1 | TMPOP2 | -4.6 | 3.3.E-03 |
| ENSG00000172819.17 | RARG | -4.6 | 2.7.E-03 |
| ENSG00000187775.17 | DNAH17 | -4.6 | 2.0.E-04 |
| ENSG00000180432.6 | CYP8B1 | -4.5 | 2.7.E-03 |
| ENSG00000128284.19 | APOL3 | -4.5 | 2.7.E-03 |
