# Supplementary material for: Comprehensive transcriptome profiling of BET inhibitor-treated HepG2 cells
Source: PLoS One. 2022 Apr 29;17(4):e0266966. doi: 10.1371/journal.pone.0266966 (PMC9053788; doi:10.1371/journal.pone.0266966)
Supplement: S4 Table — (DOCX) [file pone.0266966.s010.docx]

**S4 Table. Top 50 significant up- and downregulated DEmRNAs in OTX015-treated HepG2 cells.**

| **Ensembl_id** | **mRNA_symbol** | **Log2FoldChange** | ***p*adj** |
| --- | --- | --- | --- |
| ENSG00000113657.13 | DPYSL3 | 6.2 | 2.5.E-10 |
| ENSG00000084710.14 | EFR3B | 5 | 6.1.E-51 |
| ENSG00000197380.11 | DACT3 | 4.5 | 4.2.E-03 |
| ENSG00000267385.1 | AC011498.4 | 4.5 | 6.2.E-03 |
| ENSG00000173805.16 | HAP1 | 4.3 | 1.8.E-03 |
| ENSG00000103056.12 | SMPD3 | 4.2 | 2.2.E-03 |
| ENSG00000163220.11 | S100A9 | 4.2 | 1.4.E-03 |
| ENSG00000101098.13 | RIMS4 | 4.1 | 1.1.E-03 |
| ENSG00000179399.15 | GPC5 | 4.1 | 2.6.E-02 |
| ENSG00000163995.21 | ABLIM2 | 4 | 6.1.E-03 |
| ENSG00000170629.14 | DPY19L2P2 | 3.8 | 1.4.E-03 |
| ENSG00000201944.1 | SNORA72 | 3.6 | 3.8.E-02 |
| ENSG00000199730.1 | RN7SKP95 | 3.6 | 2.5.E-02 |
| ENSG00000172817.4 | CYP7B1 | 3.4 | 7.0.E-04 |
| ENSG00000170921.15 | TANC2 | 3.4 | 1.4.E-02 |
| ENSG00000198576.4 | ARC | 3.4 | 2.4.E-02 |
| ENSG00000283676.1 | MIR5087 | 3.4 | 3.7.E-02 |
| ENSG00000196376.11 | SLC35F1 | 3.4 | 3.1.E-02 |
| ENSG00000155367.15 | PPM1J | 3.4 | 2.6.E-02 |
| ENSG00000144369.13 | FAM171B | 3.4 | 2.5.E-03 |
| ENSG00000100027.17 | YPEL1 | 3.3 | 6.9.E-05 |
| ENSG00000154917.11 | RAB6B | 3.3 | 4.7.E-02 |
| ENSG00000101298.15 | SNPH | 3.2 | 2.1.E-02 |
| ENSG00000182759.4 | MAFA | 3.2 | 8.8.E-03 |
| ENSG00000149260.18 | CAPN5 | 3.1 | 5.2.E-06 |
| ENSG00000139971.15 | ARMH4 | 3.1 | 4.4.E-02 |
| ENSG00000166750.10 | SLFN5 | 3.1 | 4.8.E-02 |
| ENSG00000241120.1 | HMGN1P8 | 3.1 | 2.4.E-02 |
| ENSG00000145198.14 | VWA5B2 | 3 | 1.9.E-03 |
| ENSG00000170345.10 | FOS | 3 | 5.0.E-10 |
| ENSG00000278952.1 | AP003068.4 | 3 | 4.9.E-02 |
| ENSG00000183508.5 | TENT5C | 2.9 | 3.4.E-02 |
| ENSG00000166963.13 | MAP1A | 2.9 | 1.0.E-02 |
| ENSG00000145147.20 | SLIT2 | 2.9 | 3.2.E-02 |
| ENSG00000121753.13 | ADGRB2 | 2.9 | 4.3.E-02 |
| ENSG00000148798.11 | INA | 2.8 | 5.0.E-03 |
| ENSG00000214456.8 | PLIN5 | 2.7 | 3.4.E-05 |
| ENSG00000207005.1 | RNU1-2 | 2.7 | 2.4.E-05 |
| ENSG00000125266.8 | EFNB2 | 2.7 | 1.7.E-02 |
| ENSG00000206828.1 | RNVU1-30 | 2.7 | 4.7.E-05 |
| ENSG00000223203.1 | RNA5SP221 | 2.6 | 8.4.E-06 |
| ENSG00000135709.12 | KIAA0513 | 2.6 | 5.2.E-08 |
| ENSG00000130775.16 | THEMIS2 | 2.6 | 1.1.E-04 |
| ENSG00000286172.1 | RNVU1-8 | 2.6 | 9.2.E-04 |
| ENSG00000248121.8 | SMURF2P1 | 2.6 | 2.4.E-02 |
| ENSG00000265972.6 | TXNIP | 2.5 | 4.7.E-27 |
| ENSG00000127415.13 | IDUA | 2.5 | 5.2.E-04 |
| ENSG00000154146.13 | NRGN | 2.5 | 8.6.E-05 |
| ENSG00000100867.15 | DHRS2 | 2.5 | 1.7.E-37 |
| ENSG00000106003.13 | LFNG | 2.4 | 5.6.E-10 |
| ENSG00000074211.14 | PPP2R2C | -6.3 | 3.6.E-06 |
| ENSG00000172482.5 | AGXT | -6.2 | 2.0.E-05 |
| ENSG00000112337.11 | SLC17A2 | -6 | 1.3.E-05 |
| ENSG00000005981.13 | ASB4 | -6 | 1.9.E-05 |
| ENSG00000165376.12 | CLDN2 | -6 | 1.8.E-05 |
| ENSG00000177669.4 | MBOAT4 | -5.8 | 1.6.E-04 |
| ENSG00000167656.5 | LY6D | -5.7 | 1.9.E-04 |
| ENSG00000249853.8 | HS3ST5 | -5.5 | 2.3.E-04 |
| ENSG00000164406.8 | LEAP2 | -5.5 | 1.1.E-04 |
| ENSG00000172572.7 | PDE3A | -5.3 | 3.7.E-04 |
| ENSG00000136167.15 | LCP1 | -5.3 | 2.4.E-04 |
| ENSG00000121075.11 | TBX4 | -5.3 | 8.6.E-04 |
| ENSG00000172478.18 | MAB21L4 | -5.3 | 7.7.E-04 |
| ENSG00000123609.11 | NMI | -5.2 | 2.6.E-04 |
| ENSG00000012504.15 | NR1H4 | -5.2 | 2.8.E-32 |
| ENSG00000132274.16 | TRIM22 | -5.2 | 8.6.E-04 |
| ENSG00000188959.10 | C9orf152 | -5.2 | 6.7.E-04 |
| ENSG00000101082.14 | SLA2 | -5.2 | 1.2.E-03 |
| ENSG00000100024.15 | UPB1 | -5.1 | 1.1.E-03 |
| ENSG00000197558.13 | SSPOP | -5.1 | 9.0.E-04 |
| ENSG00000154016.14 | GRAP | -5.1 | 2.1.E-03 |
| ENSG00000183145.9 | RIPPLY3 | -5 | 1.4.E-03 |
| ENSG00000159625.15 | DRC7 | -5 | 1.6.E-03 |
| ENSG00000125895.5 | TMEM74B | -5 | 6.8.E-04 |
| ENSG00000243955.6 | GSTA1 | -4.9 | 1.1.E-03 |
| ENSG00000196917.6 | HCAR1 | -4.9 | 3.0.E-03 |
| ENSG00000177984.7 | LCN15 | -4.9 | 4.5.E-03 |
| ENSG00000130427.3 | EPO | -4.8 | 2.5.E-03 |
| ENSG00000149742.10 | SLC22A9 | -4.8 | 3.2.E-03 |
| ENSG00000132965.10 | ALOX5AP | -4.8 | 5.9.E-03 |
| ENSG00000213197.3 | NUDCP1 | -4.7 | 4.4.E-03 |
| ENSG00000185615.16 | PDIA2 | -4.7 | 4.4.E-03 |
| ENSG00000127241.18 | MASP1 | -4.6 | 2.6.E-03 |
| ENSG00000161653.11 | NAGS | -4.6 | 2.0.E-03 |
| ENSG00000180432.6 | CYP8B1 | -4.5 | 3.1.E-03 |
| ENSG00000114737.16 | CISH | -4.5 | 5.0.E-03 |
| ENSG00000133800.9 | LYVE1 | -4.5 | 3.2.E-03 |
| ENSG00000203985.11 | LDLRAD1 | -4.5 | 3.6.E-03 |
| ENSG00000132530.17 | XAF1 | -4.5 | 2.8.E-03 |
| ENSG00000112494.10 | UNC93A | -4.5 | 4.3.E-04 |
| ENSG00000212901.4 | KRTAP3-1 | -4.5 | 1.7.E-02 |
| ENSG00000258818.4 | RNASE4 | -4.4 | 8.7.E-03 |
| ENSG00000124602.10 | UNC5CL | -4.4 | 5.6.E-04 |
| ENSG00000182156.10 | ENPP7 | -4.4 | 4.0.E-03 |
| ENSG00000252316.1 | RNY4 | -4.4 | 3.9.E-02 |
| ENSG00000146755.11 | TRIM50 | -4.3 | 5.6.E-04 |
| ENSG00000155792.10 | DEPTOR | -4.3 | 6.8.E-03 |
| ENSG00000163131.12 | CTSS | -4.3 | 1.4.E-02 |
| ENSG00000144130.11 | NT5DC4 | -4.3 | 2.8.E-02 |
| ENSG00000131477.11 | RAMP2 | -4.3 | 1.5.E-02 |
