# Supplementary material for: Comprehensive transcriptome profiling of BET inhibitor-treated HepG2 cells
Source: PLoS One. 2022 Apr 29;17(4):e0266966. doi: 10.1371/journal.pone.0266966 (PMC9053788; doi:10.1371/journal.pone.0266966)
Supplement: S5 Table — (DOCX) [file pone.0266966.s011.docx]

**S5 Table. Top 50 significant up- and downregulated DElncRNAs in OTX015-treated HepG2 cells.**

| **Ensembl_id** | **lncRNA_symbol** | **Log2FoldChange** | ***p*adj** |
| --- | --- | --- | --- |
| ENSG00000287064.1 | AL606500.1 | 4.9 | 3.4.E-49 |
| ENSG00000265800.1 | AC022211.3 | 3.8 | 2.6.E-03 |
| ENSG00000287529.1 | AC068946.3 | 3.6 | 5.7.E-08 |
| ENSG00000272468.1 | AL021807.1 | 3.5 | 4.6.E-02 |
| ENSG00000259523.1 | AC022613.2 | 3.4 | 4.2.E-02 |
| ENSG00000188825.14 | LINC00910 | 3.3 | 3.3.E-60 |
| ENSG00000259033.2 | AL356804.1 | 3.0 | 1.7.E-03 |
| ENSG00000276216.1 | AC245014.3 | 2.7 | 7.6.E-06 |
| ENSG00000287979.1 | AC253572.1 | 2.6 | 6.7.E-09 |
| ENSG00000245532.9 | NEAT1 | 2.5 | 1.2.E-20 |
| ENSG00000235903.9 | CPB2-AS1 | 2.3 | 4.1.E-04 |
| ENSG00000286176.2 | AC116317.1 | 2.3 | 2.7.E-03 |
| ENSG00000224043.8 | CCNT2-AS1 | 2.2 | 8.2.E-11 |
| ENSG00000285646.2 | AL021155.4 | 2.2 | 3.6.E-12 |
| ENSG00000249790.3 | AC092490.1 | 2.1 | 5.8.E-14 |
| ENSG00000262202.4 | AC007952.4 | 2.1 | 2.5.E-02 |
| ENSG00000270022.3 | Z93241.1 | 2.1 | 1.0.E-04 |
| ENSG00000278916.1 | CEP83-DT | 2.0 | 4.3.E-02 |
| ENSG00000272079.2 | AC004233.2 | 1.9 | 2.4.E-03 |
| ENSG00000287190.1 | AC239799.2 | 1.9 | 7.1.E-04 |
| ENSG00000286403.1 | AC010378.1 | 1.9 | 9.3.E-38 |
| ENSG00000261324.2 | AC010168.2 | 1.7 | 3.0.E-05 |
| ENSG00000247095.3 | MIR210HG | 1.7 | 1.4.E-02 |
| ENSG00000205559.5 | CHKB-DT | 1.6 | 2.6.E-05 |
| ENSG00000285796.1 | AL162458.1 | 1.5 | 6.5.E-19 |
| ENSG00000231131.8 | LNCAROD | -7.2 | 2.2.E-07 |
| ENSG00000231826.6 | LINC01819 | -5.7 | 3.2.E-04 |
| ENSG00000264404.3 | LINC02675 | -5.3 | 7.5.E-04 |
| ENSG00000269976.1 | AC012065.2 | -5.3 | 9.9.E-04 |
| ENSG00000246526.2 | LINC02481 | -5.2 | 1.5.E-03 |
| ENSG00000248461.3 | LINC02119 | -5.2 | 2.4.E-03 |
| ENSG00000287828.1 | AL354743.2 | -5.1 | 2.6.E-03 |
| ENSG00000286117.2 | AL121894.3 | -5.0 | 2.7.E-03 |
| ENSG00000253196.1 | AC083841.1 | -5.0 | 8.1.E-03 |
| ENSG00000261058.1 | AC099508.2 | -4.9 | 6.3.E-03 |
| ENSG00000255650.6 | FAM222A-AS1 | -4.8 | 1.0.E-02 |
| ENSG00000253686.2 | LINC01484 | -4.8 | 1.4.E-02 |
| ENSG00000231574.6 | LINC02015 | -4.6 | 1.8.E-02 |
| ENSG00000276867.1 | AC074050.4 | -4.5 | 7.8.E-03 |
| ENSG00000286101.1 | AC093894.2 | -4.5 | 2.9.E-02 |
| ENSG00000236095.1 | AL807757.2 | -4.5 | 4.1.E-02 |
| ENSG00000267675.1 | AC105105.2 | -4.4 | 4.0.E-02 |
| ENSG00000260963.2 | AC026462.3 | -4.4 | 1.3.E-02 |
| ENSG00000259376.1 | AC090907.1 | -4.4 | 4.2.E-02 |
| ENSG00000233392.5 | UICLM | -4.4 | 1.6.E-03 |
| ENSG00000286715.1 | AL589863.2 | -4.4 | 4.1.E-02 |
| ENSG00000231172.2 | AC007099.1 | -4.3 | 2.3.E-03 |
| ENSG00000286011.1 | AC062037.3 | -4.2 | 4.2.E-02 |
| ENSG00000255745.2 | AC023796.1 | -4.2 | 4.2.E-02 |
| ENSG00000257817.2 | AC026765.3 | -4.1 | 2.9.E-02 |
| ENSG00000266402.3 | SNHG25 | -4.1 | 3.4.E-02 |
| ENSG00000238164.6 | TNFRSF14-AS1 | -4.0 | 3.9.E-02 |
| ENSG00000262558.1 | AC129507.3 | -4.0 | 3.4.E-02 |
| ENSG00000265254.1 | AC015917.2 | -3.9 | 4.5.E-02 |
| ENSG00000276649.1 | AL117335.1 | -3.9 | 4.5.E-02 |
| ENSG00000230303.6 | AL353743.2 | -3.9 | 4.3.E-02 |
| ENSG00000253522.6 | MIR3142HG | -3.8 | 5.3.E-07 |
| ENSG00000268926.3 | AL354861.3 | -3.6 | 2.7.E-03 |
| ENSG00000254166.3 | CASC19 | -3.5 | 8.5.E-33 |
| ENSG00000243083.7 | LINC00870 | -3.5 | 4.1.E-02 |
| ENSG00000248727.6 | LINC01948 | -3.3 | 1.2.E-04 |
| ENSG00000257345.2 | LINC02413 | -3.2 | 1.1.E-08 |
| ENSG00000258867.6 | LINC01146 | -3.2 | 1.5.E-18 |
| ENSG00000234155.1 | LINC02535 | -3.1 | 1.3.E-03 |
| ENSG00000229214.2 | LINC00242 | -3.0 | 9.0.E-07 |
| ENSG00000231566.2 | LINC02595 | -2.9 | 4.2.E-02 |
| ENSG00000254290.1 | AC124067.4 | -2.8 | 8.2.E-11 |
| ENSG00000197085.11 | NPSR1-AS1 | -2.8 | 2.0.E-13 |
| ENSG00000288100.1 | AL161663.2 | -2.7 | 8.1.E-03 |
| ENSG00000224417.2 | AL606970.1 | -2.6 | 2.6.E-03 |
| ENSG00000286271.2 | AC008945.2 | -2.5 | 8.2.E-04 |
| ENSG00000260604.2 | AL590004.3 | -2.5 | 3.0.E-09 |
| ENSG00000229005.2 | HNF4A-AS1 | -2.4 | 8.0.E-05 |
| ENSG00000231948.2 | HS1BP3-IT1 | -2.3 | 2.7.E-05 |
| ENSG00000237361.3 | TUSC8 | -2.3 | 5.5.E-05 |
