# Supplementary material for: Comprehensive transcriptome profiling of BET inhibitor-treated HepG2 cells
Source: PLoS One. 2022 Apr 29;17(4):e0266966. doi: 10.1371/journal.pone.0266966 (PMC9053788; doi:10.1371/journal.pone.0266966)
Supplement: S6 Table — (DOCX) [file pone.0266966.s012.docx]

**S6 Table. Top 50 significant up- and downregulated DEmRNAs in ABBV-075-treated HepG2 cells.**

| **Ensembl_id** | **mRNA_symbol** | **Log2FoldChange** | ***p*adj** |
| --- | --- | --- | --- |
| ENSG00000113657.13 | DPYSL3 | 6.7 | 1.6.E-12 |
| ENSG00000084710.14 | EFR3B | 5.4 | 1.8.E-59 |
| ENSG00000103056.12 | SMPD3 | 5 | 5.3.E-05 |
| ENSG00000197380.11 | DACT3 | 4.6 | 2.1.E-03 |
| ENSG00000173805.16 | HAP1 | 4.3 | 1.2.E-03 |
| ENSG00000116991.11 | SIPA1L2 | 4.2 | 4.4.E-03 |
| ENSG00000103196.12 | CRISPLD2 | 4.2 | 9.4.E-04 |
| ENSG00000173868.12 | PHOSPHO1 | 4.1 | 2.0.E-02 |
| ENSG00000166750.10 | SLFN5 | 4 | 2.8.E-03 |
| ENSG00000232466.1 | H3P31 | 4 | 9.6.E-03 |
| ENSG00000267385.1 | AC011498.4 | 4 | 1.4.E-02 |
| ENSG00000163995.21 | ABLIM2 | 3.9 | 6.1.E-03 |
| ENSG00000280407.2 | AC132872.4 | 3.9 | 4.5.E-03 |
| ENSG00000145147.20 | SLIT2 | 3.8 | 1.1.E-03 |
| ENSG00000149260.18 | CAPN5 | 3.8 | 2.2.E-09 |
| ENSG00000095587.9 | TLL2 | 3.7 | 5.3.E-03 |
| ENSG00000101098.13 | RIMS4 | 3.7 | 3.0.E-03 |
| ENSG00000227766.1 | AL671277.1 | 3.6 | 4.2.E-02 |
| ENSG00000206848.1 | RNU6-890P | 3.6 | 3.5.E-02 |
| ENSG00000167646.14 | DNAAF3 | 3.6 | 3.0.E-02 |
| ENSG00000283676.1 | MIR5087 | 3.6 | 1.8.E-02 |
| ENSG00000280430.1 | AP002782.1 | 3.5 | 3.3.E-02 |
| ENSG00000214456.8 | PLIN5 | 3.5 | 4.2.E-09 |
| ENSG00000226632.1 | UBE2V1P1 | 3.5 | 2.2.E-02 |
| ENSG00000144369.13 | FAM171B | 3.5 | 1.0.E-03 |
| ENSG00000121101.16 | TEX14 | 3.4 | 3.5.E-02 |
| ENSG00000279464.1 | AC096720.2 | 3.4 | 4.0.E-02 |
| ENSG00000170629.14 | DPY19L2P2 | 3.4 | 3.8.E-03 |
| ENSG00000155367.15 | PPM1J | 3.4 | 1.9.E-02 |
| ENSG00000005249.13 | PRKAR2B | 3.4 | 4.1.E-02 |
| ENSG00000170921.15 | TANC2 | 3.3 | 1.4.E-02 |
| ENSG00000100027.17 | YPEL1 | 3.3 | 4.1.E-05 |
| ENSG00000229992.1 | HMGB3P9 | 3.2 | 3.9.E-02 |
| ENSG00000183508.5 | TENT5C | 3.2 | 1.2.E-02 |
| ENSG00000154917.11 | RAB6B | 3.1 | 4.8.E-02 |
| ENSG00000241120.1 | HMGN1P8 | 3.1 | 1.4.E-02 |
| ENSG00000278952.1 | AP003068.4 | 3.1 | 2.8.E-02 |
| ENSG00000166963.13 | MAP1A | 3 | 4.9.E-03 |
| ENSG00000196376.11 | SLC35F1 | 3 | 4.8.E-02 |
| ENSG00000145198.14 | VWA5B2 | 3 | 1.2.E-03 |
| ENSG00000135709.12 | KIAA0513 | 3 | 4.0.E-11 |
| ENSG00000183793.14 | NPIPA5 | 3 | 4.0.E-02 |
| ENSG00000125266.8 | EFNB2 | 2.9 | 4.6.E-03 |
| ENSG00000182759.4 | MAFA | 2.9 | 1.4.E-02 |
| ENSG00000223203.1 | RNA5SP221 | 2.9 | 2.0.E-07 |
| ENSG00000158246.8 | TENT5B | 2.9 | 3.6.E-02 |
| ENSG00000265972.6 | TXNIP | 2.9 | 6.9.E-36 |
| ENSG00000120329.7 | SLC25A2 | 2.9 | 3.5.E-02 |
| ENSG00000172817.4 | CYP7B1 | 2.9 | 5.0.E-03 |
| ENSG00000206828.1 | RNVU1-30 | 2.8 | 9.8.E-06 |
| ENSG00000112337.11 | SLC17A2 | -6.4 | 2.4.E-06 |
| ENSG00000082074.19 | FYB1 | -6.1 | 3.5.E-02 |
| ENSG00000012504.15 | NR1H4 | -5.7 | 2.1.E-35 |
| ENSG00000023171.18 | GRAMD1B | -5.6 | 5.7.E-04 |
| ENSG00000132274.16 | TRIM22 | -5.5 | 2.4.E-04 |
| ENSG00000168065.16 | SLC22A11 | -5.4 | 4.9.E-04 |
| ENSG00000198650.11 | TAT | -5.4 | 3.8.E-06 |
| ENSG00000128573.26 | FOXP2 | -5.3 | 1.1.E-02 |
| ENSG00000205918.9 | PDPK2P | -5.3 | 1.3.E-03 |
| ENSG00000248763.2 | AC111000.2 | -5.2 | 2.5.E-03 |
| ENSG00000106991.14 | ENG | -5 | 2.8.E-03 |
| ENSG00000283162.1 | AL390726.5 | -5 | 1.5.E-03 |
| ENSG00000113249.13 | HAVCR1 | -5 | 7.4.E-04 |
| ENSG00000135097.7 | MSI1 | -4.9 | 2.4.E-03 |
| ENSG00000180116.15 | C12orf40 | -4.9 | 8.1.E-03 |
| ENSG00000180432.6 | CYP8B1 | -4.9 | 9.7.E-04 |
| ENSG00000102230.14 | PCYT1B | -4.9 | 6.3.E-27 |
| ENSG00000165376.12 | CLDN2 | -4.8 | 1.4.E-06 |
| ENSG00000212901.4 | KRTAP3-1 | -4.8 | 7.3.E-03 |
| ENSG00000172572.7 | PDE3A | -4.7 | 1.5.E-03 |
| ENSG00000198133.8 | TMEM229B | -4.7 | 7.5.E-03 |
| ENSG00000123405.14 | NFE2 | -4.6 | 5.3.E-08 |
| ENSG00000121075.11 | TBX4 | -4.6 | 3.0.E-03 |
| ENSG00000197599.12 | CCDC154 | -4.6 | 2.3.E-02 |
| ENSG00000160183.17 | TMPRSS3 | -4.6 | 2.5.E-03 |
| ENSG00000146216.13 | TTBK1 | -4.5 | 3.3.E-03 |
| ENSG00000086730.17 | LAT2 | -4.5 | 9.0.E-03 |
| ENSG00000188959.10 | C9orf152 | -4.5 | 2.6.E-03 |
| ENSG00000156413.15 | FUT6 | -4.5 | 5.0.E-12 |
| ENSG00000064201.16 | TSPAN32 | -4.5 | 4.0.E-02 |
| ENSG00000227437.1 | RPS8P4 | -4.5 | 3.9.E-02 |
| ENSG00000074211.14 | PPP2R2C | -4.4 | 1.4.E-08 |
| ENSG00000139269.3 | INHBE | -4.4 | 1.0.E-14 |
| ENSG00000177679.16 | SRRM3 | -4.4 | 1.2.E-02 |
| ENSG00000027075.15 | PRKCH | -4.4 | 1.0.E-02 |
| ENSG00000125895.5 | TMEM74B | -4.4 | 4.6.E-04 |
| ENSG00000183117.19 | CSMD1 | -4.4 | 1.6.E-02 |
| ENSG00000170324.21 | FRMPD2 | -4.3 | 1.9.E-02 |
| ENSG00000172482.5 | AGXT | -4.3 | 2.4.E-06 |
| ENSG00000164406.8 | LEAP2 | -4.3 | 2.2.E-05 |
| ENSG00000140067.6 | FAM181A | -4.3 | 4.2.E-03 |
| ENSG00000266217.2 | CTSLP2 | -4.3 | 2.5.E-02 |
| ENSG00000169507.9 | SLC38A11 | -4.3 | 1.8.E-06 |
| ENSG00000064787.13 | BCAS1 | -4.3 | 5.9.E-03 |
| ENSG00000156096.14 | UGT2B4 | -4.3 | 1.1.E-05 |
| ENSG00000167656.5 | LY6D | -4.3 | 2.3.E-03 |
| ENSG00000218996.1 | ARL4AP5 | -4.2 | 1.4.E-02 |
| ENSG00000157514.16 | TSC22D3 | -4.2 | 1.6.E-02 |
| ENSG00000126785.13 | RHOJ | -4.2 | 5.7.E-03 |
| ENSG00000112494.10 | UNC93A | -4.2 | 4.4.E-05 |
