# Supplementary material for: Comprehensive transcriptome profiling of BET inhibitor-treated HepG2 cells
Source: PLoS One. 2022 Apr 29;17(4):e0266966. doi: 10.1371/journal.pone.0266966 (PMC9053788; doi:10.1371/journal.pone.0266966)
Supplement: S7 Table — (DOCX) [file pone.0266966.s013.docx]

**S7 Table. Top 50 significant up- and downregulated DElncRNAs in ABBV-075-treated HepG2 cells.**

| **Ensembl_id** | **lncRNA_symbol** | **Log2FoldChange** | ***p*adj** |
| --- | --- | --- | --- |
| ENSG00000287064.1 | AL606500.1 | 5.1 | 2.3.E-53 |
| ENSG00000235643.2 | LINC01647 | 4.3 | 1.8.E-03 |
| ENSG00000265800.1 | AC022211.3 | 4.0 | 7.0.E-04 |
| ENSG00000287529.1 | AC068946.3 | 3.6 | 1.2.E-08 |
| ENSG00000188825.14 | LINC00910 | 3.6 | 7.7.E-72 |
| ENSG00000259523.1 | AC022613.2 | 3.4 | 2.8.E-02 |
| ENSG00000259033.2 | AL356804.1 | 3.3 | 1.8.E-04 |
| ENSG00000276216.1 | AC245014.3 | 3.0 | 7.7.E-08 |
| ENSG00000269906.1 | AL606834.1 | 2.8 | 1.7.E-02 |
| ENSG00000245532.9 | NEAT1 | 2.7 | 1.1.E-25 |
| ENSG00000287979.1 | AC253572.1 | 2.6 | 7.2.E-09 |
| ENSG00000235903.9 | CPB2-AS1 | 2.6 | 3.4.E-05 |
| ENSG00000286176.2 | AC116317.1 | 2.5 | 2.1.E-04 |
| ENSG00000227210.2 | AC079145.1 | 2.4 | 1.5.E-02 |
| ENSG00000276255.2 | LINC02809 | 2.4 | 3.4.E-02 |
| ENSG00000285646.2 | AL021155.4 | 2.3 | 2.8.E-14 |
| ENSG00000224043.8 | CCNT2-AS1 | 2.3 | 2.0.E-12 |
| ENSG00000262202.4 | AC007952.4 | 2.3 | 5.2.E-03 |
| ENSG00000249790.3 | AC092490.1 | 2.1 | 4.9.E-14 |
| ENSG00000270022.3 | Z93241.1 | 2.1 | 9.0.E-05 |
| ENSG00000287190.1 | AC239799.2 | 2.1 | 7.9.E-05 |
| ENSG00000278916.1 | CEP83-DT | 2.0 | 2.1.E-02 |
| ENSG00000228393.4 | LINC01004 | 2.0 | 3.2.E-03 |
| ENSG00000261324.2 | AC010168.2 | 2.0 | 2.5.E-07 |
| ENSG00000286403.1 | AC010378.1 | 2.0 | 2.6.E-43 |
| ENSG00000272079.2 | AC004233.2 | 1.9 | 1.3.E-03 |
| ENSG00000272054.1 | AC007390.1 | 1.8 | 2.9.E-02 |
| ENSG00000237037.9 | NDUFA6-DT | 1.7 | 1.8.E-02 |
| ENSG00000274422.1 | AC245060.5 | 1.6 | 4.6.E-02 |
| ENSG00000205559.5 | CHKB-DT | 1.6 | 7.3.E-06 |
| ENSG00000285796.1 | AL162458.1 | 1.6 | 6.8.E-22 |
| ENSG00000272405.1 | AL365181.3 | 1.6 | 3.6.E-07 |
| ENSG00000253671.3 | AC027117.1 | 1.6 | 6.3.E-09 |
| ENSG00000225975.7 | LINC01534 | 1.6 | 3.4.E-02 |
| ENSG00000276337.1 | AC105429.1 | 1.5 | 1.5.E-04 |
| ENSG00000247095.3 | MIR210HG | 1.5 | 2.9.E-02 |
| ENSG00000231131.8 | LNCAROD | -5.4 | 1.3.E-05 |
| ENSG00000227712.2 | AL359915.1 | -5.4 | 8.4.E-04 |
| ENSG00000287828.1 | AL354743.2 | -5.2 | 1.6.E-03 |
| ENSG00000260620.1 | AC027688.2 | -5.0 | 3.4.E-03 |
| ENSG00000261058.1 | AC099508.2 | -4.9 | 3.5.E-03 |
| ENSG00000265254.1 | AC015917.2 | -4.9 | 3.4.E-03 |
| ENSG00000255650.6 | FAM222A-AS1 | -4.9 | 5.6.E-03 |
| ENSG00000266401.2 | AP002478.1 | -4.9 | 3.4.E-03 |
| ENSG00000267123.7 | SCAT1 | -4.8 | 5.2.E-03 |
| ENSG00000231826.6 | LINC01819 | -4.8 | 3.2.E-03 |
| ENSG00000253522.6 | MIR3142HG | -4.4 | 5.9.E-07 |
| ENSG00000248461.3 | LINC02119 | -4.2 | 1.5.E-02 |
| ENSG00000286117.2 | AL121894.3 | -4.2 | 4.3.E-03 |
| ENSG00000236412.1 | AC092941.2 | -4.2 | 1.5.E-02 |
| ENSG00000254166.3 | CASC19 | -4.0 | 7.7.E-34 |
| ENSG00000237361.3 | TUSC8 | -3.8 | 2.0.E-06 |
| ENSG00000269976.1 | AC012065.2 | -3.8 | 1.3.E-03 |
| ENSG00000197085.11 | NPSR1-AS1 | -3.7 | 1.1.E-15 |
| ENSG00000258867.6 | LINC01146 | -3.7 | 1.3.E-19 |
| ENSG00000229214.2 | LINC00242 | -3.7 | 1.6.E-07 |
| ENSG00000260963.2 | AC026462.3 | -3.6 | 1.9.E-02 |
| ENSG00000257345.2 | LINC02413 | -3.6 | 3.0.E-09 |
| ENSG00000286714.1 | AC093001.2 | -3.5 | 2.0.E-03 |
| ENSG00000231948.2 | HS1BP3-IT1 | -3.5 | 2.9.E-07 |
| ENSG00000264404.3 | LINC02675 | -3.4 | 9.2.E-04 |
| ENSG00000241388.5 | HNF1A-AS1 | -3.4 | 6.7.E-11 |
| ENSG00000245648.2 | KLRK1-AS1 | -3.3 | 3.9.E-02 |
| ENSG00000231172.2 | AC007099.1 | -3.3 | 1.0.E-03 |
| ENSG00000254290.1 | AC124067.4 | -3.2 | 6.6.E-12 |
| ENSG00000288100.1 | AL161663.2 | -3.1 | 3.0.E-03 |
| ENSG00000229425.3 | AJ009632.2 | -3.0 | 4.3.E-03 |
| ENSG00000286271.2 | AC008945.2 | -2.9 | 1.9.E-04 |
| ENSG00000260604.2 | AL590004.3 | -2.9 | 6.7.E-11 |
| ENSG00000246526.2 | LINC02481 | -2.8 | 3.9.E-02 |
| ENSG00000224189.8 | HAGLR | -2.7 | 1.0.E-29 |
| ENSG00000229056.2 | HECW2-AS1 | -2.7 | 1.7.E-02 |
| ENSG00000248727.6 | LINC01948 | -2.7 | 1.5.E-04 |
| ENSG00000285517.1 | AC010198.2 | -2.7 | 3.9.E-02 |
| ENSG00000234753.5 | FOXP4-AS1 | -2.7 | 3.4.E-02 |
| ENSG00000233392.5 | UICLM | -2.6 | 1.7.E-03 |
| ENSG00000234155.1 | LINC02535 | -2.6 | 1.7.E-03 |
| ENSG00000268926.3 | AL354861.3 | -2.6 | 3.8.E-03 |
| ENSG00000246334.2 | PRR7-AS1 | -2.6 | 2.7.E-03 |
| ENSG00000274979.1 | AC020656.2 | -2.6 | 5.3.E-21 |
| ENSG00000152931.9 | PART1 | -2.6 | 4.0.E-07 |
| ENSG00000286733.1 | LINC02348 | -2.5 | 1.4.E-03 |
| ENSG00000282221.1 | AC119427.1 | -2.5 | 1.5.E-02 |
| ENSG00000229005.2 | HNF4A-AS1 | -2.4 | 3.7.E-05 |
| ENSG00000261713.6 | SSTR5-AS1 | -2.4 | 2.2.E-02 |
| ENSG00000285872.1 | AC007240.3 | -2.4 | 3.6.E-03 |
