# Supplementary material for: Comprehensive transcriptome profiling of BET inhibitor-treated HepG2 cells
Source: PLoS One. 2022 Apr 29;17(4):e0266966. doi: 10.1371/journal.pone.0266966 (PMC9053788; doi:10.1371/journal.pone.0266966)
Supplement: S8 Table — (DOCX) [file pone.0266966.s014.docx]

**S8 Table. Top 50 significant up- and downregulated DEmRNAs in BET inhibitor-treated HepG2 cells in common.**

| **mRNA_symbol** | **JQ1** | | **OTX-015** | | **ABBV-075** | |
| --- | --- | --- | --- | --- | --- | --- |
|  | Log2FC | *p*adj | Log2FC | *p*adj | Log2FC | *p*adj |
| DPYSL3 | 6.1 | 2.9.E-10 | 6.2 | 2.5.E-10 | 6.7 | 1.6.E-12 |
| EFR3B | 5.1 | 2.7.E-52 | 5 | 6.1.E-51 | 5.4 | 1.8.E-59 |
| SMPD3 | 4.7 | 2.9.E-04 | 4.2 | 2.2.E-03 | 5 | 5.3.E-05 |
| SLIT2 | 3.9 | 1.1.E-03 | 2.9 | 3.2.E-02 | 3.8 | 1.1.E-03 |
| SLFN5 | 3.9 | 5.5.E-03 | 3.1 | 4.8.E-02 | 4 | 2.8.E-03 |
| DACT3 | 3.8 | 2.2.E-02 | 4.5 | 4.2.E-03 | 4.6 | 2.1.E-03 |
| CAPN5 | 3.8 | 5.5.E-09 | 3.1 | 5.2.E-06 | 3.8 | 2.2.E-09 |
| ABLIM2 | 3.7 | 1.3.E-02 | 4 | 6.1.E-03 | 3.9 | 6.1.E-03 |
| MIR5087 | 3.7 | 1.5.E-02 | 3.4 | 3.7.E-02 | 3.6 | 1.8.E-02 |
| PLIN5 | 3.5 | 6.1.E-09 | 2.7 | 3.4.E-05 | 3.5 | 4.2.E-09 |
| DPY19L2P2 | 3.5 | 3.5.E-03 | 3.8 | 1.4.E-03 | 3.4 | 3.8.E-03 |
| SLC35F1 | 3.4 | 2.9.E-02 | 3.4 | 3.1.E-02 | 3 | 4.8.E-02 |
| HAP1 | 3.1 | 3.6.E-02 | 4.3 | 1.8.E-03 | 4.3 | 1.2.E-03 |
| VWA5B2 | 3 | 1.3.E-03 | 3 | 1.9.E-03 | 3 | 1.2.E-03 |
| FAM171B | 3 | 8.6.E-03 | 3.4 | 2.5.E-03 | 3.5 | 1.0.E-03 |
| AP003068.4 | 3 | 4.9.E-02 | 3 | 4.9.E-02 | 3.1 | 2.8.E-02 |
| YPEL1 | 2.9 | 7.0.E-04 | 3.3 | 6.9.E-05 | 3.3 | 4.1.E-05 |
| HMGN1P8 | 2.8 | 4.1.E-02 | 3.1 | 2.4.E-02 | 3.1 | 1.4.E-02 |
| MAP1A | 2.8 | 1.4.E-02 | 2.9 | 1.0.E-02 | 3 | 4.9.E-03 |
| KIAA0513 | 2.8 | 1.3.E-09 | 2.6 | 5.2.E-08 | 3 | 4.0.E-11 |
| TENT5C | 2.8 | 4.5.E-02 | 2.9 | 3.4.E-02 | 3.2 | 1.2.E-02 |
| TXNIP | 2.8 | 1.3.E-32 | 2.5 | 4.7.E-27 | 2.9 | 6.9.E-36 |
| IDUA | 2.7 | 5.9.E-05 | 2.5 | 5.2.E-04 | 2.8 | 2.7.E-05 |
| RNA5SP221 | 2.7 | 4.5.E-06 | 2.6 | 8.4.E-06 | 2.9 | 2.0.E-07 |
| RNVU1-2 | 2.6 | 4.7.E-05 | 2 | 4.4.E-03 | 2.4 | 1.5.E-04 |
| RNVU1-30 | 2.6 | 1.3.E-04 | 2.7 | 4.7.E-05 | 2.8 | 9.8.E-06 |
| DHRS2 | 2.4 | 2.0.E-37 | 2.5 | 1.7.E-37 | 2.7 | 2.6.E-45 |
| EFNB2 | 2.4 | 4.0.E-02 | 2.7 | 1.7.E-02 | 2.9 | 4.6.E-03 |
| ARRDC4 | 2.4 | 3.9.E-14 | 2.3 | 2.3.E-13 | 2.5 | 1.2.E-16 |
| ABCA3 | 2.4 | 1.1.E-04 | 1.9 | 5.4.E-03 | 2.3 | 1.4.E-04 |
| AC093323.1 | 2.4 | 2.0.E-30 | 2.4 | 9.0.E-32 | 2.6 | 1.7.E-37 |
| ZC3H6 | 2.3 | 8.6.E-09 | 2.3 | 1.0.E-08 | 2.5 | 1.5.E-10 |
| FOS | 2.3 | 3.6.E-06 | 3 | 5.0.E-10 | 2.8 | 5.6.E-09 |
| RNU1-2 | 2.3 | 5.9.E-04 | 2.7 | 2.4.E-05 | 2.7 | 9.6.E-06 |
| PAG1 | 2.3 | 2.1.E-11 | 2 | 1.4.E-08 | 2.4 | 3.9.E-13 |
| CYP7B1 | 2.3 | 4.5.E-02 | 3.4 | 7.0.E-04 | 2.9 | 5.0.E-03 |
| GEMIN8P4 | 2.3 | 3.3.E-02 | 2.3 | 3.6.E-02 | 2.4 | 1.9.E-02 |
| ZNF117 | 2.3 | 3.9.E-06 | 2 | 4.9.E-05 | 2.3 | 1.8.E-06 |
| TMEM175 | 2.2 | 1.1.E-05 | 1.8 | 6.5.E-04 | 2.2 | 7.8.E-06 |
| INA | 2.2 | 4.2.E-02 | 2.8 | 5.0.E-03 | 2.7 | 5.5.E-03 |
| ABCA5 | 2.1 | 4.4.E-13 | 1.9 | 4.0.E-10 | 2.2 | 3.1.E-14 |
| DSEL | 2.1 | 3.3.E-03 | 2.3 | 9.7.E-04 | 2.4 | 2.7.E-04 |
| ZC3H10 | 2.1 | 6.6.E-17 | 2.3 | 3.3.E-20 | 2.4 | 1.4.E-23 |
| RNVU1-3 | 2.1 | 6.6.E-03 | 1.9 | 2.3.E-02 | 2 | 6.5.E-03 |
| KCNC3 | 2.1 | 9.6.E-06 | 1.7 | 7.6.E-04 | 2.1 | 2.8.E-06 |
| AC109326.1 | 2 | 4.2.E-27 | 2 | 6.3.E-27 | 2.2 | 1.0.E-32 |
| ZNF837 | 2 | 2.2.E-05 | 1.8 | 2.1.E-04 | 2.1 | 2.7.E-06 |
| G6PD | 2 | 1.5.E-22 | 2.1 | 1.8.E-24 | 2.2 | 2.0.E-28 |
| SESN3 | 2 | 1.2.E-11 | 2 | 2.3.E-11 | 2.1 | 2.8.E-14 |
| DOP1B | 2 | 2.3.E-12 | 1.8 | 7.0.E-11 | 2.1 | 1.2.E-14 |
| SLC17A2 | -7 | 2.3.E-07 | -6 | 1.3.E-05 | -6.4 | 2.4.E-07 |
| C8A | -6.6 | 2.2.E-06 | -3.1 | 1.7.E-04 | -4.1 | 1.4.E-06 |
| UNC93A | -6.3 | 5.1.E-06 | -4.5 | 4.3.E-04 | -4.2 | 5.8.E-06 |
| NFE2 | -6.2 | 4.8.E-06 | -4 | 5.2.E-07 | -4.6 | 3.8.E-09 |
| TRIM50 | -6.2 | 6.9.E-06 | -4.3 | 5.6.E-04 | -4.1 | 8.1.E-06 |
| LGALS2 | -6.1 | 1.6.E-05 | -2.3 | 6.0.E-03 | -3 | 7.3.E-05 |
| TMEM74B | -5.9 | 2.8.E-05 | -5 | 6.8.E-04 | -4.4 | 8.0.E-05 |
| LY6D | -5.7 | 1.7.E-04 | -5.7 | 1.9.E-04 | -4.3 | 5.0.E-04 |
| PLCH2 | -5.7 | 8.6.E-05 | -2.5 | 1.2.E-02 | -3.1 | 3.5.E-04 |
| TSPAN1 | -5.6 | 2.5.E-04 | -2.4 | 3.3.E-02 | -3.5 | 9.6.E-04 |
| HAVCR1 | -5.6 | 1.4.E-04 | -3.2 | 8.4.E-03 | -5 | 1.4.E-04 |
| NAGS | -5.6 | 1.1.E-04 | -4.6 | 2.0.E-03 | -3.4 | 3.2.E-04 |
| ITGA10 | -5.3 | 3.2.E-04 | -3.5 | 1.3.E-02 | -2.8 | 2.0.E-03 |
| TBX4 | -5.3 | 7.4.E-04 | -5.3 | 8.6.E-04 | -4.6 | 6.7.E-04 |
| MAB21L4 | -5.3 | 6.7.E-04 | -5.3 | 7.7.E-04 | -3.7 | 2.1.E-03 |
| GDPD3 | -5.2 | 3.1.E-08 | -1.8 | 3.9.E-06 | -3 | 4.1.E-13 |
| TRIM22 | -5.2 | 7.5.E-04 | -5.2 | 8.6.E-04 | -5.5 | 3.9.E-05 |
| MAB21L3 | -5.1 | 1.1.E-03 | -2.6 | 1.4.E-02 | -2.7 | 1.6.E-03 |
| CLDN2 | -5.1 | 3.1.E-05 | -6 | 1.8.E-05 | -4.8 | 1.3.E-07 |
| GRAP | -5.1 | 1.9.E-03 | -5.1 | 2.1.E-03 | -3.5 | 5.5.E-03 |
| RHBDL2 | -5 | 7.3.E-04 | -3.2 | 2.6.E-02 | -2.9 | 3.4.E-03 |
| PCYT1B | -5 | 1.1.E-21 | -4 | 7.2.E-24 | -4.9 | 4.5.E-29 |
| POU2AF1 | -5 | 1.4.E-03 | -4.1 | 1.4.E-02 | -3.5 | 4.5.E-03 |
| RIPPLY3 | -5 | 1.3.E-03 | -5 | 1.4.E-03 | -2.9 | 6.1.E-03 |
| DRC7 | -5 | 1.4.E-03 | -5 | 1.6.E-03 | -2.9 | 6.7.E-03 |
| ALDH8A1 | -5 | 8.0.E-05 | -1.7 | 1.1.E-02 | -2.7 | 1.3.E-05 |
| GGT1 | -4.9 | 7.6.E-04 | -2 | 1.9.E-02 | -3.4 | 1.1.E-04 |
| AC111000.2 | -4.9 | 5.9.E-03 | -3.9 | 3.6.E-02 | -5.2 | 5.4.E-04 |
| AIFM3 | -4.8 | 6.8.E-05 | -1.5 | 7.3.E-03 | -2.5 | 3.1.E-06 |
| SECTM1 | -4.8 | 1.2.E-03 | -1.9 | 3.8.E-02 | -3.3 | 2.8.E-04 |
| EPO | -4.8 | 2.2.E-03 | -4.8 | 2.5.E-03 | -4.2 | 2.0.E-03 |
| ITIH1 | -4.7 | 3.0.E-03 | -3.8 | 2.5.E-02 | -2.6 | 1.6.E-02 |
| AL390726.5 | -4.7 | 3.8.E-03 | -3.7 | 3.0.E-02 | -5 | 2.9.E-04 |
| NR1H4 | -4.6 | 1.6.E-34 | -5.2 | 2.8.E-32 | -5.7 | 9.1.E-38 |
| DNAH17 | -4.6 | 2.0.E-04 | -4 | 1.2.E-04 | -3.6 | 1.3.E-06 |
| CYP8B1 | -4.5 | 2.7.E-03 | -4.5 | 3.1.E-03 | -4.9 | 1.8.E-04 |
| APOL3 | -4.5 | 2.7.E-03 | -3.6 | 8.8.E-03 | -2.9 | 9.7.E-04 |
| KRTAP3-1 | -4.5 | 1.6.E-02 | -4.5 | 1.7.E-02 | -4.8 | 1.8.E-03 |
| PPP2R2C | -4.4 | 3.9.E-07 | -6.3 | 3.6.E-06 | -4.4 | 8.9.E-10 |
| LCP1 | -4.4 | 5.3.E-04 | -5.3 | 2.4.E-04 | -4.1 | 1.0.E-05 |
| CCDC154 | -4.2 | 4.2.E-02 | -4.2 | 4.5.E-02 | -4.6 | 6.7.E-03 |
| TMPRSS3 | -4.2 | 6.4.E-03 | -3.3 | 2.0.E-02 | -4.6 | 5.3.E-04 |
| INHA | -4.2 | 8.7.E-05 | -1.9 | 5.6.E-03 | -2.6 | 2.6.E-05 |
| PSMB10 | -4.1 | 7.1.E-03 | -3.2 | 2.3.E-02 | -2.2 | 7.6.E-03 |
| INHBE | -4.1 | 1.6.E-12 | -3.5 | 2.2.E-12 | -4.4 | 2.6.E-16 |
| TAT | -4.1 | 2.9.E-06 | -3.5 | 2.7.E-06 | -5.4 | 3.9.E-07 |
| SRRM3 | -4.1 | 2.4.E-02 | -4.1 | 2.6.E-02 | -4.4 | 3.1.E-03 |
| FRMPD2 | -4 | 3.7.E-02 | -4 | 3.9.E-02 | -4.3 | 5.4.E-03 |
| IL1RN | -4 | 5.5.E-10 | -3.8 | 6.2.E-10 | -4 | 3.1.E-13 |
| AKR1D1 | -3.9 | 2.9.E-10 | -4.1 | 4.0.E-10 | -4 | 1.0.E-13 |
