# Supplementary material for: Comprehensive transcriptome profiling of BET inhibitor-treated HepG2 cells
Source: PLoS One. 2022 Apr 29;17(4):e0266966. doi: 10.1371/journal.pone.0266966 (PMC9053788; doi:10.1371/journal.pone.0266966)
Supplement: S9 Table — (DOCX) [file pone.0266966.s015.docx]

**S9 Table. Top 50 significant up- and downregulated DElncRNAs in BET inhibitor-treated HepG2 cells in common.**

| **lncRNA_symbol** | **JQ1** | | **OTX-015** | | **ABBV-075** | |
| --- | --- | --- | --- | --- | --- | --- |
|  | Log2FC | *p*adj | Log2FC | *p*adj | Log2FC | *p*adj |
| AL606500.1 | 4.6 | 4.3.E-43 | 4.9 | 3.4.E-49 | 5.1 | 2.3.E-53 |
| LINC00910 | 3.2 | 2.0.E-56 | 3.3 | 3.3.E-60 | 3.6 | 7.7.E-72 |
| AC022211.3 | 3.2 | 2.2.E-02 | 3.8 | 2.6.E-03 | 4.0 | 7.0.E-04 |
| AC068946.3 | 3.1 | 5.2.E-06 | 3.6 | 5.7.E-08 | 3.6 | 1.2.E-08 |
| AL356804.1 | 3.0 | 1.5.E-03 | 3.0 | 1.7.E-03 | 3.3 | 1.8.E-04 |
| AC245014.3 | 2.9 | 7.5.E-07 | 2.7 | 7.6.E-06 | 3.0 | 7.7.E-08 |
| NEAT1 | 2.6 | 8.4.E-23 | 2.5 | 1.2.E-20 | 2.7 | 1.1.E-25 |
| CPB2-AS1 | 2.5 | 1.7.E-04 | 2.3 | 4.1.E-04 | 2.6 | 3.4.E-05 |
| AC116317.1 | 2.3 | 2.2.E-03 | 2.3 | 2.7.E-03 | 2.5 | 2.1.E-04 |
| AL021155.4 | 2.1 | 7.5.E-11 | 2.2 | 3.6.E-12 | 2.3 | 2.8.E-14 |
| CCNT2-AS1 | 2.1 | 2.1.E-09 | 2.2 | 8.2.E-11 | 2.3 | 2.0.E-12 |
| AC007952.4 | 2.1 | 2.4.E-02 | 2.1 | 2.5.E-02 | 2.3 | 5.2.E-03 |
| AC253572.1 | 2.0 | 8.0.E-05 | 2.6 | 6.7.E-09 | 2.6 | 7.2.E-09 |
| AC092490.1 | 2.0 | 1.2.E-11 | 2.1 | 5.8.E-14 | 2.1 | 4.9.E-14 |
| AC010168.2 | 1.8 | 7.3.E-06 | 1.7 | 3.0.E-05 | 2.0 | 2.5.E-07 |
| AC004233.2 | 1.8 | 5.5.E-03 | 1.9 | 2.4.E-03 | 1.9 | 1.3.E-03 |
| AC010378.1 | 1.8 | 7.5.E-35 | 1.9 | 9.3.E-38 | 2.0 | 2.6.E-43 |
| AC239799.2 | 1.7 | 4.2.E-03 | 1.9 | 7.1.E-04 | 2.1 | 7.9.E-05 |
| CHKB-DT | 1.6 | 3.2.E-05 | 1.6 | 2.6.E-05 | 1.6 | 7.3.E-06 |
| AC012065.2 | -6.2 | 4.2.E-05 | -5.3 | 9.9.E-04 | -3.8 | 1.3.E-03 |
| AL121894.3 | -6.0 | 1.6.E-04 | -5.0 | 2.7.E-03 | -4.2 | 4.3.E-03 |
| LINC01819 | -5.7 | 3.4.E-04 | -5.7 | 3.2.E-04 | -4.8 | 3.2.E-03 |
| AC026462.3 | -5.4 | 8.8.E-04 | -4.4 | 1.3.E-02 | -3.6 | 1.9.E-02 |
| LNCAROD | -5.4 | 2.5.E-05 | -7.2 | 2.2.E-07 | -5.4 | 1.3.E-05 |
| LINC02481 | -5.3 | 1.5.E-03 | -5.2 | 1.5.E-03 | -2.8 | 3.9.E-02 |
| AL354743.2 | -5.1 | 2.6.E-03 | -5.1 | 2.6.E-03 | -5.2 | 1.6.E-03 |
| TUSC8 | -5.1 | 5.6.E-05 | -2.3 | 5.5.E-05 | -3.8 | 2.0.E-06 |
| AC093001.2 | -5.0 | 1.6.E-03 | -1.9 | 3.2.E-02 | -3.5 | 2.0.E-03 |
| AL354861.3 | -4.2 | 3.4.E-03 | -3.6 | 2.7.E-03 | -2.6 | 3.8.E-03 |
| MIR3142HG | -4.1 | 7.3.E-07 | -3.8 | 5.3.E-07 | -4.4 | 5.9.E-07 |
| AC015917.2 | -3.9 | 4.3.E-02 | -3.9 | 4.5.E-02 | -4.9 | 3.4.E-03 |
| AL161663.2 | -3.5 | 4.2.E-03 | -2.7 | 8.1.E-03 | -3.1 | 3.0.E-03 |
| NPSR1-AS1 | -3.4 | 5.1.E-15 | -2.8 | 2.0.E-13 | -3.7 | 1.1.E-15 |
| CASC19 | -3.3 | 6.4.E-32 | -3.5 | 8.5.E-33 | -4.0 | 7.7.E-34 |
| HNF1A-AS1 | -3.1 | 3.6.E-10 | -2.1 | 2.3.E-07 | -3.4 | 6.7.E-11 |
| HS1BP3-IT1 | -3.0 | 1.7.E-06 | -2.3 | 2.7.E-05 | -3.5 | 2.9.E-07 |
| LINC01146 | -3.0 | 5.6.E-18 | -3.2 | 1.5.E-18 | -3.7 | 1.3.E-19 |
| AC124067.4 | -2.9 | 4.7.E-11 | -2.8 | 8.2.E-11 | -3.2 | 6.6.E-12 |
| PRR7-AS1 | -2.9 | 3.2.E-03 | -1.8 | 2.9.E-02 | -2.6 | 2.7.E-03 |
| LINC02413 | -2.8 | 3.5.E-08 | -3.2 | 1.1.E-08 | -3.6 | 3.0.E-09 |
| LINC00242 | -2.7 | 2.1.E-06 | -3.0 | 9.0.E-07 | -3.7 | 1.6.E-07 |
| LINC02675 | -2.6 | 4.6.E-03 | -5.3 | 7.5.E-04 | -3.4 | 9.2.E-04 |
| AL590004.3 | -2.5 | 1.6.E-09 | -2.5 | 3.0.E-09 | -2.9 | 6.7.E-11 |
| LINC01948 | -2.5 | 4.7.E-04 | -3.3 | 1.2.E-04 | -2.7 | 1.5.E-04 |
| AC008945.2 | -2.4 | 1.5.E-03 | -2.5 | 8.2.E-04 | -2.9 | 1.9.E-04 |
| AC020656.2 | -2.3 | 1.7.E-17 | -2.1 | 3.2.E-16 | -2.6 | 5.3.E-21 |
| HAGLR | -2.2 | 4.9.E-23 | -2.3 | 2.0.E-24 | -2.7 | 1.0.E-29 |
| LINC02348 | -2.2 | 5.5.E-03 | -2.0 | 1.1.E-02 | -2.5 | 1.4.E-03 |
| UICLM | -2.1 | 1.1.E-02 | -4.4 | 1.6.E-03 | -2.6 | 1.7.E-03 |
| AC007240.3 | -2.0 | 1.6.E-02 | -2.0 | 1.8.E-02 | -2.4 | 3.6.E-03 |
| LINC02535 | -2.0 | 1.5.E-02 | -3.1 | 1.3.E-03 | -2.6 | 1.7.E-03 |
| AJ009632.2 | -2.0 | 3.8.E-02 | -2.1 | 3.0.E-02 | -3.0 | 4.3.E-03 |
| AC099344.1 | -1.9 | 8.0.E-12 | -1.5 | 1.4.E-08 | -2.2 | 1.7.E-14 |
| LINC00261 | -1.9 | 3.3.E-12 | -2.0 | 5.8.E-13 | -2.3 | 4.7.E-16 |
| PART1 | -1.9 | 5.8.E-05 | -2.2 | 6.2.E-06 | -2.6 | 4.0.E-07 |
| AC007099.1 | -1.8 | 2.6.E-02 | -4.3 | 2.3.E-03 | -3.3 | 1.0.E-03 |
| AL606970.1 | -1.8 | 2.1.E-02 | -2.6 | 2.6.E-03 | -2.4 | 2.2.E-03 |
| AC007493.1 | -1.7 | 1.7.E-18 | -2.2 | 1.5.E-25 | -2.3 | 2.5.E-28 |
| HNF4A-AS1 | -1.7 | 3.3.E-03 | -2.4 | 8.0.E-05 | -2.4 | 3.7.E-05 |
| LINC01348 | -1.5 | 3.9.E-05 | -1.6 | 1.3.E-05 | -2.0 | 1.1.E-07 |
